# T-Cell-Specific CerS4 Depletion Prolonged Inflammation and Enhanced Tumor Burden in the AOM/DSS-Induced CAC Model

**DOI:** 10.3390/ijms23031866

**Published:** 2022-02-07

**Authors:** Khadija El-Hindi, Sebastian Brachtendorf, Jennifer Christina Hartel, Stephanie Oertel, Kerstin Birod, Nadine Merz, Sandra Trautmann, Dominique Thomas, Andreas Weigert, Tim J. Schäufele, Klaus Scholich, Susanne Schiffmann, Thomas Ulshöfer, Olaf Utermöhlen, Sabine Grösch

**Affiliations:** 1 Institute of Clinical Pharmacology, Goethe-University Frankfurt, 60590 Frankfurt, Germany; El-Hindi@med.uni-frankfurt.de (K.E.-H.); Sebastian.Brachtendorf@web.de (S.B.); jhartel@med.uni-frankfurt.de (J.C.H.); stephi1106@hotmail.com (S.O.); k.birod@med.uni-frankfurt.de (K.B.); N.Merz@med.uni-frankfurt.de (N.M.); trautmann@med.uni-frankfurt.de (S.T.); thomas@med.uni-frankfurt.de (D.T.); schaeufele@med.uni-frankfurt.de (T.J.S.); scholich@em.uni-frankfurt.de (K.S.); 2 Department of Life Sciences, Goethe-University Frankfurt, 60590 Frankfurt, Germany; 3 Fraunhofer Institute for Translational Medicine and Pharmacology ITMP, 60596 Frankfurt, Germany; susanne.schiffmann@itmp.fraunhofer.de (S.S.); thomas.ulshoefer@itmp.fraunhofer.de (T.U.); 4 Institute of Biochemistry I, Faculty of Medicine, Goethe-University Frankfurt, 60590 Frankfurt, Germany; weigert@biochem.uni-frankfurt.de; 5 Center for Molecular Medicine Cologne, Institute for Medical Microbiology, Immunology and Hygiene, University of Cologne, 50935 Cologne, Germany; olaf.utermoehlen@uk-koeln.de

**Keywords:** ceramide synthase, LASS, colon, tumor, T-cell, Jurkat

## Abstract

To better understand the role of sphingolipids in the multifactorial process of inflammatory bowel disease (IBD), we elucidated the role of CerS4 in colitis and colitis-associated cancer (CAC). For this, we utilized the azoxymethane/dextran sodium sulphate (AOM/DSS)-induced colitis model in global CerS4 knockout (CerS4 KO), intestinal epithelial (CerS4 Vil/Cre), or T-cell restricted knockout (CerS4 LCK/Cre) mice. CerS4 KO mice were highly sensitive to the toxic effect of AOM/DSS, leading to a high mortality rate. CerS4 Vil/Cre mice had smaller tumors than WT mice. In contrast, CerS4 LCK/Cre mice frequently suffered from pancolitis and developed more colon tumors. In vitro, CerS4-depleted CD8+ T-cells isolated from the thymi of CerS4 LCK/Cre mice showed impaired proliferation and prolonged cytokine production after stimulation in comparison with T-cells from WT mice. Depletion of CerS4 in human Jurkat T-cells led to a constitutively activated T-cell receptor and NF-κB signaling pathway. In conclusion, the deficiency of CerS4 in T-cells led to an enduring active status of these cells and prevents the resolution of inflammation, leading to a higher tumor burden in the CAC mouse model. In contrast, CerS4 deficiency in epithelial cells resulted in smaller colon tumors and seemed to be beneficial. The higher tumor incidence in CerS4 LCK/Cre mice and the toxic effect of AOM/DSS in CerS4 KO mice exhibited the importance of CerS4 in other tissues and revealed the complexity of general targeting CerS4.

## 1. Introduction

Although the overall incidence and mortality of colorectal cancers has decreased over the last ten years, the incidence in younger people (<50 years) has increased [1,2]. It is supposed that the increase has resulted from a shift in various lifestyle factors, such as diet and physical activity. In this context, it fits that the incidence and prevalence of inflammatory bowel disease, including Crohn’s disease (CD) and ulcerative colitis (UC), and especially the incidence in younger people, has risen over time [3]. IBD enhances the risk for colitis-associated colon cancer (CAC), which depends on the disease severity (extensive colorectal inflammation) and duration [4,5,6]. Both colon cancer and IBD are associated with changes in the microbiome [7] and energy, amino acid, and lipid metabolism [8,9,10,11].

Among the lipid alterations in colitis patients, sphingolipids of various chain lengths are changed [12,13]. Sphingolipids act not only as important membrane components but as second messengers. They influence cellular processes such as apoptosis, cell proliferation and differentiation, migration, and signaling of various growth factors such as the insulin-like growth factor. Therefore, they play important roles under physiological and pathophysiological conditions [14,15,16]. Alterations in sphingolipid levels can occur by either deregulation of de novo synthesis or a disturbed salvage pathway. In both colitis and colorectal cancer, de novo synthesis is dysregulated, implying that this key step in sphingolipid de novo synthesis is involved in the pathophysiology of IBD [12,17]. Central enzymes to sphingolipid de novo synthesis are ceramide synthases (CerSs), formerly also known as the longevity assurance homologue (Lass) gene family, which are responsible for the synthesis of dihydroceramides or ceramides through acetylation of sphinganine or sphingosine with a fatty acyl CoA moiety of different chain length. To date, six CerSs are known in mammalians. They differ in their chain length specificity, facilitating a defined regulation of chain length-specific synthesis of ceramides (Cer). Long chain ceramides are produced by CerS1 (C18-Cer), CerS4 (C18-Cer and C20-Cer), and CerS5 and CerS6 (C14-Cer and C16-Cer), and very long chain ceramides are produced by CerS2 (C22-Cer, C24-Cer) and CerS3 (up to C34-Cer).

Previous studies revealed the impact of distinct CerSs in colitis and CAC models. CerS2 and very long chain Cer are important for intestinal barrier function [18]. C16:0-Cer is the dominant ceramide in the colon produced by CerS5 and CerS6. Although both CerS6 KO and CerS5 KO mice suffered from enhanced inflammation, the mechanisms behind this phenomenon differed and were tissue specific. While CerS6 KO mice showed remarkably enhanced inflammation depending on increased infiltration of neutrophils into the colon [19], an adoptive transfer of CerS6-deficient splenocytes resulted in reduced intestinal inflammation [20]. CerS5 is more important for immune regulation than for intestinal epithelial cell function. Hence, a global CerS5 knockdown resulted in a reduced number of intestinal CD8^+^ T-cells, which are important for intestinal homeostasis and inflammatory regulation, but the lack of CerS5 in epithelial cells did not worsen disease progression [21]. These studies highlighted the importance of CerSs in the inflammatory process of UC. CerS4 is downregulated in both UC patients and colorectal cancer patients [12,17]. To investigate the influence of CerS4 in colitis and CAC, we analyzed CerS4-deficient mice in a dextran sodium sulfate (DSS) and azoxymethane (AOM)/DSS model. Based on previous findings, we also differentiated the tissue-specific impact of CerS4 by including intestinal epithelial CerS4 knockout (CerS4 Vil1/Cre) and T-cell-specific CerS4 knockout (CerS4 LCK/Cre).

## 2. Results

### 2.1. Tissue-Specific Impact of CerS4 Deficiency in the AOM/DSS CAC Model

**Figure 1:**
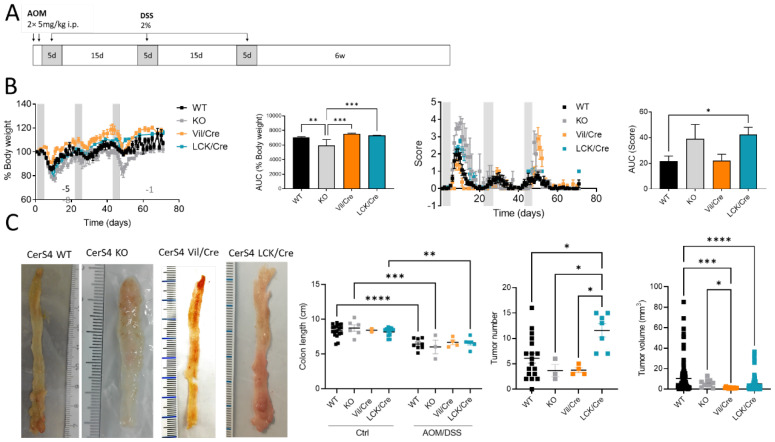
Effect of tissue-specific CerS4 deficiency in a colitis-associated cancer model. (**A**) Treatment schemata of AOM/DSS model: on the first 2 days, 5 mg/kg AOM was injected intraperitoneally (i.p.), and inflammation was induced by three cycles of 2% DSS in the drinking water for 5 days after 15 days recovery. After 6 weeks, the mice were killed. (**B**) Disease was monitored of CerS4 WT, CerS4 KO, CerS4 Vil/Cre, and CerS4 LCK/Cre during AOM/DSS treatment via either behavior of body weight or score until day 71 with the calculated area under the curve (AUC). The AUC was calculated from all mice reaching the end of the experiment. (**C**) At the end of the experiment, colon length and tumor development were investigated. The representative pictures show the colons of AOM/DSS-treated mice. The colon lengths of AOM/DSS-treated mice were reduced in comparison with those of control mice. Tumor number and volume were quantified by macroscopic evaluation. Data are mean ±SEM. Statistical analysis was performed by two-way ANOVA (C; colon length) or one-way ANOVA (**B**,**C**). Group sizes of control mice: WT, *n* = 18; KO, *n* = 9; Vil/Cre, *n* = 2; and LCK/Cre, *n* = 12. Group sizes for AOM/DSS-treated mice: WT, *n* = 23; KO, *n* = 12; Vil/Cre, *n* = 4; and LCK/Cre, *n* = 7. * *p* < 0.05, ** *p* < 0.01, *** *p* < 0.001, **** *p* < 0.0001.

We investigated the tissue-specific impact of CerS4 deficiency in the AOM/DSS CAC model, using complete CerS4 knockout mice (CerS4 KO) but also tissue-specific knockouts in which the CerS4 deficiency was restricted either to the intestinal epithelium (Vil/Cre) or to the T-cells (LCK/Cre). To mimic CAC, the mice were treated with AOM/DSS. AOM is a mutagenic compound that accelerates colon tumor development, especially when it is applied in combination with repeated administration of DSS, leading to epithelial damage and inflammation in the colon (Figure 1A). CerS4 WT mice responded to DSS by the loss of body weight and symptoms of colitis such as diarrhea, bloody stool, and colon tumors 6 weeks after the last DSS cycle. However, the CerS4-deficient mice responded differently from the WT mice (Figure 1B). Complete CerS4-deficient mice (CerS4 KO) had considerable body weight loss after AOM injection and during the first cycle of DSS, leading to an early abortion of the experiment in 62% of the CerS4 KO mice in comparison with 13% of the CerS4 WT mice. The body weight losses of WT, CerS4 Vil/Cre, and CerS4 LCK/Cre mice were not as severe as those of CerS4 KO mice. CerS4 LCK/Cre mice exhibited higher sensitivity to AOM/DSS treatment than WT mice (Figure 1B). All treated mice except CerS4 Vil/Cre mice had significant shortening of the colon compared with their respective control groups (Figure 1C). Interestingly, CerS4 LCK/Cre mice developed significantly more colon tumors than CerS4 WT, CerS4 KO, and CerS4 Vil/Cre mice. Although the tumor number was higher in AOM/DSS-treated CerS4 LCK/Cre mice, the tumor volume was lower than in WT mice (Figure 1C).

**Figure 2:**
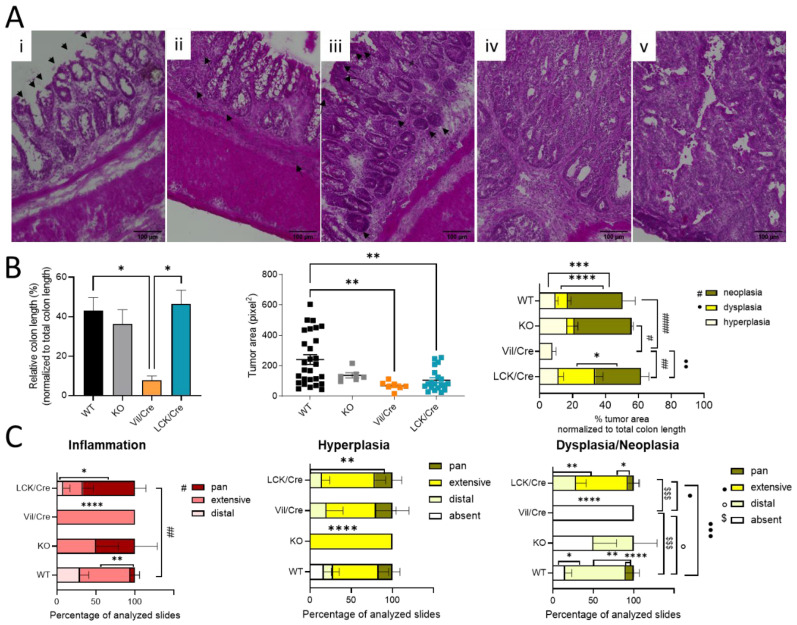
Histological analysis of colon rolls. (**A**) A hematoxylin and eosin-stained colon was taken with BIOREVO (Keyence) (20× lens). Inflammation was indicated by (**i**) destructed crypts (indicated by black arrows) and (**ii**) thicker lamina propria filled with immune cells (indicated by black arrows) and a thicker muscularis. Tumor growth starts with (**iii**) hyperplasia, which is marked by hyperchromatic regions and elongated nuclei (indicated by black arrows). Hyperplasia might lead to a nonreversible (**iv**) dysplasia ending with (**v**) a neoplasia. Scale bars (100 µm) were added with FIJI. (**B**) Quantitative analysis with FIJI: percentage of tumor area related to whole colon length. Measuring the tumor area indicated bigger tumors in WT mice. Percentages of tumor tissue (hyperplasia, dysplasia, and neoplasia) in relation to colon length were also calculated. (**C**) The dispersion of inflamed, hyperplasic, and dysplastic areas in the colon was determined in different sections and divided into distal, extensive (about half of the colon length), or pan (whole colon). The percentage of disease spreading in all analyzed slices are shown. Data are mean ±SEM. Statistical analysis was performed by one-way ANOVA and two-way ANOVA for Figure 2B,C, respectively, with a multiple comparison test: (* *p* < 0.05, ** *p* < 0.01, *** *p* < 0.001, **** *p* < 0.0001). Statistically significant differences between the different mouse strains are indicated by different symbols according to the legend. Statistically significant differences within a mouse strain are marked with stars.

To further analyze colon tissue damage and tumor development, the colon was investigated histologically. For this, Swiss rolls were stained with hematoxylin and eosin. Mostly in the distal and middle parts of the colon, inflammatory signs such as infiltrating immune cells or destructed crypts were observed. In 66% of all investigated tissue slices of CerS4 LCK/Cre mice, the inflammation extended from the distal to the proximal part of the colon (pan), while in CerS4 KO mice, about 50% of all slices exhibited pancolitis. In WT mice, only 5% of all slices showed pancolitis. In all CerS4 Vil/Cre mice, inflammatory areas were found from the distal part to the middle part of the colon (extensive) (Figure 2B,C).

Therefore, the histological analysis fit well with the score data. CerS4 KO and CerS4 LCK/Cre mice, which had the highest scores, suffered most from pancolitis. Six weeks after the last DSS cycle, tumor development was observed in all groups (Figure 2B). A closer look at the severity of the tumor growth revealed that in Vil/Cre mice, colon tumor development was limited to hyperplasia, while WT, CerS4 KO, and CerS4 LCK/Cre mice also showed dysplasia and neoplasia. The location of dysplastic or neoplastic tumors showed a similar pattern to the inflamed areas. For example, 75% of WT, 50% of CerS4 KO, and 28% of CerS4 LCK/Cre mice had tumors in the distal part of the colon (Figure 2C, right panel). The highest incidence of dysplasia was found in CerS4 LCK/Cre mice. In conclusion, CerS4 KO and CerS4 LCK/Cre mice were more susceptible to AOM/DSS-induced colitis and CAC than WT mice, whereas CerS4 Vil/Cre mice developed smaller and only preneoplastic tumors in comparison with all other groups. 

### 2.2. CerS4 KO Mice Were More Sensitive to the DSS-Induced Colitis Model

After analyzing the outcome of the mice in the chronic colitis model, we were interested to understand the inflammatory response in the colon tissue and investigated CerS4 WT, KO, and LCK/Cre in an acute DSS model. Since Vil/Cre mice showed only a mild response in the AOM/DSS model, we excluded this group for ethical reasons. 

**Figure 3:**
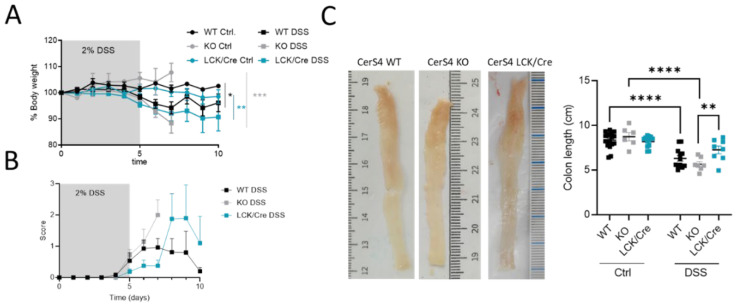
CerS4 KO, CerS4 LCK/Cre, and WT mice in the dextran sulfate sodium (DSS)-induced acute colitis model. (**A**) Reduced body weight after application of 2% DSS in drinking water for 5 days. (**B**) The disease score during treatment was not significantly different among the groups. (**C**) Representative pictures of DSS-treated colon. Quantification of colon length. DSS-treated CerS4 KO mice had significantly shorter colons than CerS4 LCK/Cre mice. Data are mean ±SEM. Statistical analysis was performed by two-way ANOVA. (* *p* < 0.05, ** *p* < 0.01, *** *p* < 0.001, **** *p* < 0.0001) Group sizes of control mice: WT, *n* = 11; KO, *n* = 6; and LCK/Cre, *n* = 12. Group sizes of DSS-treated mice: WT, *n* = 12; KO, *n* = 7; and LCK/Cre, *n* = 8.

All DSS-treated mice had a body weight drop, and their clinical disease scores increased over time (Figure 3A,B). However, in the acute inflammation model, the CerS4 KO mice and CerS4 LCK/Cre mice were most susceptible to colitis (Figure 3A). Although the scores were not significantly different, the onset of the disease was earlier in CerS4 KO than in CerS4 LCK/Cre mice (Figure 3B). The shortening of the colon is an indicator for severity of inflammation. Compared with untreated control mice, the DSS-treated mice had shorter colons, and CerS4 KO mice were again more affected than CerS4 LCK/Cre mice (Figure 3C).

In conclusion, CerS4 KO mice were more susceptible to DSS-induced inflammation than WT or CerS4 LCK/Cre mice.

### 2.3. CerS4 Depletion Affected Ceramide Level in Control and DSS-Treated Mice

**Figure 4:**
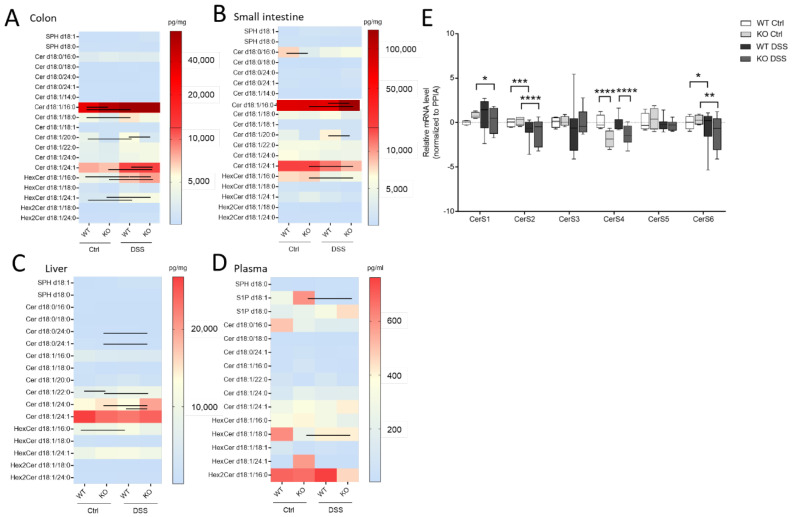
Sphingolipid and CerS expression levels in different tissues of untreated and DSS-treated CerS4 WT and KO mice. Sphingolipids in (**A**) the colon, (**B**) the small intestine, (**C**) the plasma, and (**D**) the liver were determined by LC–MS/MS. Data are medians represented in heatmaps with scale bars (pg/mg for tissue or pg/mL for plasma). Statistically significant changes between control and DSS-treated CerS4 WT and KO mice were determined by two-way ANOVA and a Tukey’s multiple comparison posttest and indicated by lines between the compared group. (**E**) CerS1–6 mRNA expression normalized to PPIA (peptidylprolyl isomerase A) in colon tissue of untreated or DSS-treated CerS4 WT and KO mice (*n* = 2–7). The relation to CerS4 WT control was calculated via relative mRNA levels as ΔΔCT (CerS4 WT Ctrl was set as 0). Statistically significant differences were determined by one-way ANOVA and a Sidak’s multiple comparison posttest (* *p* < 0.05, ** *p* < 0.01, *** *p* < 0.001, **** *p* < 0.0001). Ceramide (Cer), dihydroceramide (dhCer), glucosylceramide and galactosylceramide as hexylceramide (HexCer), lactosylceramide (dihexosylceramide, Hex2Cer).

Here, we wanted to investigate how sphingolipids, especially sphingosine, dihydro-sphingosine, and their phosphate derivatives (S1P), as well as ceramides and hexylceramides of distinct chain length, differed in the chronic or acute colitis models between the WT and CerS4 KO mice. In accordance with previous publications, C20:0-Ceramide (Cer) was reduced (*p* = 0.025, WT Ctrl: 3236 ± 676 pg/mg, *n* = 4, KO Ctrl: 998.3 ± 333 pg/mg, *n* = 4) in whole colon tissue and in the small intestine after DSS treatment (*p* = 0.0032, WT Ctrl: 3528 ± 1021 pg/mg, *n* = 3, KO Ctrl: 905.2 ± 103.8 pg/mg, *n* = 7). AOM/DSS-treated mice showed significantly reduced C20:0-Cer in the colon and liver (Figure S1A and Table S2, see Supplementary Materials). The DSS treatment seemed to amplify the sphingolipid difference between CerS4 WT and KO mice. Hence, in the DSS-treated group, statistically significant differences were observed in Cer d18:1/20:0 in both the colon and the intestine; in dihydroceramide (dhCer) d18:0/18:0 in colon tissue; and in Cer d18:1/18:0 in the small intestine (Figure 4A,B, Table S1). These data indicate that the loss of CerS4 could not be compensated by other CerSs (such as CerS1) after stress stimuli. Upon DSS-induced inflammation in the colon tissue of WT-mice, Cer d18:1/16:0, Cer d18:1/18:0, hexylceramide (HexCer; comprises glucosylceramide (GlcCer) and galactosylceramide (GalCer)) d18:1/16:0 and HexCer d18:1/24:1 increased. However, in the colon tissue of DSS-treated CerS4 KO mice, Cer d18:1/16:0, Cer d18:1/24:1, and HexCer d18:1/16:0 increased. The levels of Cer d18:1/24:1 and HexCer d18:1/16:0 dropped in the small intestine (Figure 4A,B). The increase in Cer d18:1/24:1 and HexCer d18:1/16:0 was also seen in the AOM/DSS model in the colon (Figure S1A, Table S2). Shifts in ceramide levels upon DSS treatment were also observed in liver tissue. While CerS4 WT mice exhibited elevated levels of HexCer d18:1/16:0, most differences in ceramide levels were observed in the CerS4 KO mice. The sphingolipid distribution in the liver was marked by dominant very-long-chain Cer levels (Cer d18:1/24:0 and d18:1/24:1). Long-chain Cers such as Cer d18:1/16:0 and Cer d18:1/18:0, as well as dhCer, played a minor role in liver sphingolipid composition. Like in the colon, DSS treatment induced an increase in very-long-chain sphingolipids such as dhCer d18:0/24:0 (*p* = 0.0121), dhCer d18:0/24:1 (*p* = 0.0122), Cer d18:1/22:0 (*p* = 0.0083), and Cer d18:1/24:0 (*p* = 0.0136) in CerS4 KO mice (Figure 4D). In the plasma the measured sphingolipid levels between the different mice had high variance. Hence, only S1P levels (*p* = 0.0256) dropped, and only HexCer d18:1/18:0 levels increased (*p* = 0.015), in CerS4 KO mice after DSS treatment (Figure 4C).

To investigate whether changes in the Cer levels could be attributed to changes in the expression level of CerS, the mRNA expression level of all CerS was investigated in colon tissue of WT and CerS4 KO mice that were either untreated or treated with DSS. CerS1 and CerS3 had the lowest expression level in the colons of mice, followed by CerS4, CerS5, CerS6, and CerS2. Because of the highly mixed cell composition of the tissue pieces (inflamed tissues were abundantly invaded by immune cells), the variances were high in the WT DSS-treated mice. The CerS4 expression levels in CerS4 KO mice were always significantly reduced in control and DSS-treated colon tissue in comparison with WT mice. However, no compensatory upregulation of other CerSs was observed in the control mice (CerS1 control *p* = 0.38). DSS treatment had an impact on the CerS level; in both CerS4 WT and KO mice, the expression of CerS2 and CerS6 dropped in DSS-treated in comparison with control mice. In CerS4 KO mice, CerS1 expression was also reduced upon DSS treatment, indicating that both CerS mRNA expression levels and sphingolipid levels were affected through DSS-induced inflammation in the colon tissue.

In whole-tissue samples, many cells contribute to the sphingolipid pool; hence, a CerS4 depletion in a specific cell type might not affect the sphingolipid level. Indeed, contrary to the global CerS4 KO mice, the sphingolipid profiles in the colon, liver, and plasma of Vil/Cre mice did not differ from those of WT mice (Figure S1A and Table S2). In LCK/Cre mice, the sphingolipid profiles of the thymus and spleen were analyzed. These lymphoid tissues consist of many other cells than T-cells, and hence, the sphingolipid levels were similar between WT and LCK/Cre mice. In the thymi of WT and LCK/Cre mice, HexCer d18:1/16:0 and HexCer d18:1/24:1 were decreased after DSS treatment (Figure S1B and Table S2). In the spleen, Cer d18:1/16:0, Cer d18:1/24:0 (only in WT mice), and Cer d18:1/24:1 and HexCer d18:1/24:1 (in both WT and LCK/Cre mice) were decreased (Figure S1B and Table S2). 

In summary, DSS treatment affected CerS expression in CerS4 KO and WT mice. Knockdown of CerS4 was accompanied by a significant decrease in Cer d18:0/18:0 and Cer d18:1/20:0 in the colon and small intestine. Because DSS treatment also causes changes in other sphingolipids, the extent to which CerS4 generated-sphingolipids were responsible for the higher susceptibility of CerS4 KO mice to DSS-induced colitis could not be clearly stated. 

### 2.4. CerS4 Deficiency Had No Impact on the Colon Barrier Controlled by the Tight Junction Occludin

Previous studies showed that the sphingolipid balance is important for intestinal barrier function, highlighting the importance of very-long-chain sphingolipids in barrier function [18]. Although Cer d18:1/24:1 was upregulated in CerS4 KO Ctrl mice, those mice were quite sensitive to DSS. To investigate whether the susceptibility of CerS4 KO mice to DSS-induced colitis was related to a colon barrier dysfunction, we detected the expression of tight-junction proteins occludin and zonula occludens-1 (ZO-1) in the colon tissue of CerS4 WT and CerS4 KO mice. In untreated CerS4 KO mice and CerS4 WT mice, both proteins showed clear membranous localization in epithelial cells with strong colocalization. After DSS treatment, occludin and ZO-1 vanished from the membrane and could only weakly be stained in the cytoplasm (Figure S2A). However, neither control nor DSS-treated CerS4 KO mice differed in the expression of either protein from WT mice. To strengthen these data, we extended our investigation to the human system. Therefore, CerS4 was downregulated in the human colon cell line Caco-2 by CerS4-shRNA. The barrier functionality was measured by the transepithelial electrical resistance (TEER) and the permeability of fluorescein isothiocyanate (FITC)-dextran. The electrical resistance of Caco-2 cells reached up to 800 Ωcm^2^. However, downregulation of CerS4 had no effect on the electrical resistance (Figure S2B). Moreover, the permeability of Caco-2 cells was not impaired by downregulation of CerS4 (Figure S2C). At least the immunocytochemical staining of the Caco-2 monolayer showed no difference in tight junction occludin between Caco-2 NC and Caco-2 shCerS4 cells (Figure S2D). Hence, our mice and human data indicated that CerS4 deficiency had no impact on the colon epithelial barrier function. 

### 2.5. CerS4 KO Mice Has a Greater Immune Reaction upon DSS

**Figure 5:**
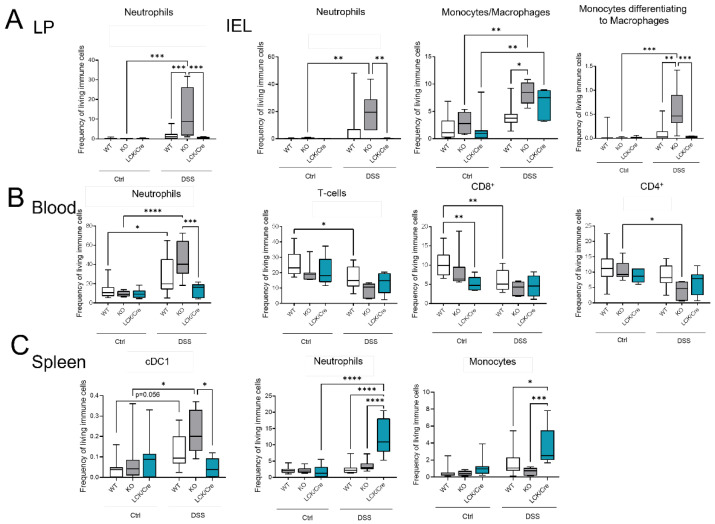
Immune cells status in mice after DSS treatment in different tissues by flow cytometry. (**A**) In the colon tissue (LP and IEL), myeloid cell (neutrophils and macrophages) levels were higher in DSS-treated CerS4 KO than in WT and LCK/Cre CerS4 mice. (**B**) In the blood, neutrophil levels were increased, and T-cells (especially CD8+) tended to be reduced, after DSS treatment. CerS4 LCK-Cre control mice already had fewer CD8+ T-cells than WT control mice before treatment. (**C**) CerS4 KO mice showed more cDC1 cells in the spleen than WT or CerS4 LCK/Cre mice. Macrophages and neutrophils were also increased in WT and LCK/Cre mice. Data plotted with median ± min to max. Statistical analysis was performed by two-way ANOVA. (* *p* < 0.05, ** *p* < 0.01, *** *p* < 0.001, **** *p* < 0.0001) Group sizes for different tissues of control mice: WT, *n* = 18–11; KO *n* = 9–5; and LCK/Cre, *n* = 9. Group sizes for different tissues of DSS-treated mice: WT, *n* = 11–12; KO, *n* = 6; and LCK/Cre, *n* = 6–8.

As CerS4 KO mice were more susceptible to acute DSS-induced colitis, we analyzed the immune cell status in the blood, spleen, and colon of these mice by flow cytometry (Figure 5). The inflammation of the colon upon DSS treatment was clearly recognizable by an increase in myeloid cells in the intraepithelial lymphocyte (IEL) fraction and lamina propria (LP) fraction. The numbers of neutrophils, monocytes, and macrophages were significantly higher in DSS-treated CerS4 KO mice than in DSS-treated CerS4 WT and CerS4 LCK/Cre mice (Figure 5A). The number of neutrophils in the blood increased in WT and CerS4 KO mice after DSS treatment, but not in CerS4 LCK/Cre mice. Contrarily, the number of T-cells decreased in WT and CerS4 KO mice after DSS treatment. However, in CerS4 LCK/Cre control mice, CD8+ T-cells were already reduced in comparison with untreated WT mice, and no further reduction was detected after DSS treatment (Figure 5B). Since the spleen filters the blood from pathogens and abnormal cells, it regulates T- and B-cell response to those antigens. In the spleen, the subpopulation of conventional DC type 1 (cDC1), with a CD8+ status on its surface and a cytotoxic response, was higher in CerS4 KO mice than in WT and CerS4 LCK/Cre mice. Contrary to that in the colon tissue and blood, the frequency of neutrophils and monocytes was higher in the spleens of CerS4 LCK/Cre mice after DSS treatment (Figure 5C). The statuses of T-cells and T-cell subpopulations were not significantly altered.

Summing up, the higher susceptibility of CerS4 KO mice to DSS-induced colitis could be confirmed by increased infiltration of neutrophils and macrophages into the colon tissue of these mice in comparison with that in WT or CerS4 LCK/Cre mice. Additionally, specific knockdown of CerS4 in T-cells reduced the number of CD8+ T-cells in control mice.

### 2.6. Different Immune Cell Status of CerS4 LCK/Cre Mice in the CAC Treatment

**Figure 6:**
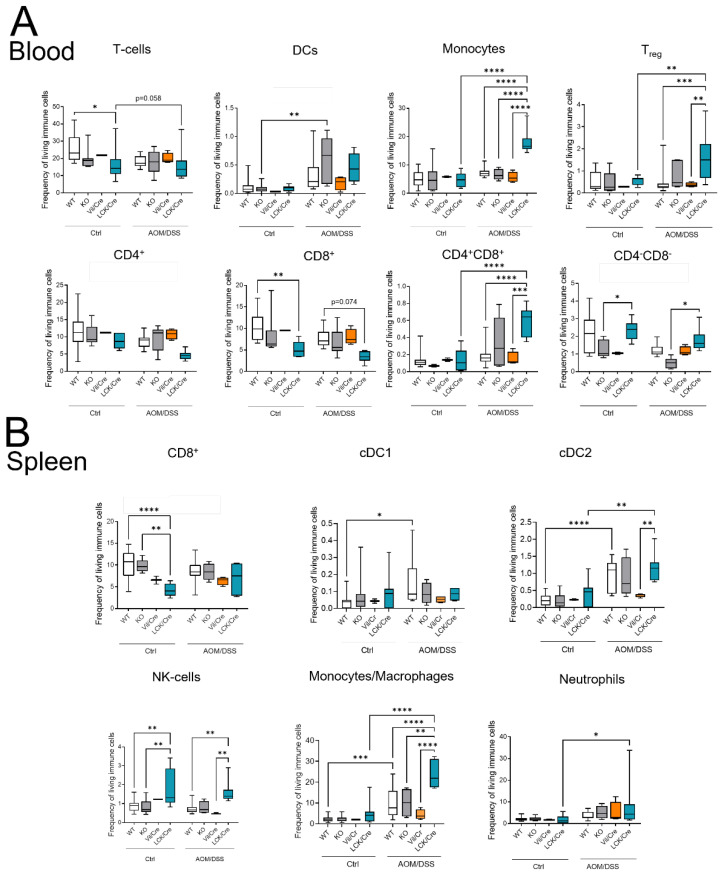
Immune cell status in mice after AOM/DSS treatment in different tissues. (**A**) Flow cytometry analysis of blood immune cells after AOM/DSS treatment showed fewer T-cells in WT and LCK/Cre mice after DSS treatment. This was based on a tendency toward lower levels of CD4+ and CD8+ T-cells. Monocytes, Tregs, and CD4+/CD8+ T-cells notably increased in AOM/DSS-treated CerS4 LCK/Cre mice. (**B**) Flow cytometry analysis of the spleen showed higher plasmacytoid DCs (pDCs) and conventional DC 2 (cDC2) in AOM/DSS-treated CerS4 LCK/Cre, whereas in AOM/DSS-treated WT and CerS4 KO mice, DC 1 (cDC1) cells notably increased. Data are plotted as medians with min to max. Statistical analysis was performed by two-way ANOVA. (* *p* < 0.05, ** *p* < 0.01, *** *p* < 0.001, **** *p* < 0.0001) Group sizes of control mice: WT, *n* = 17; KO, *n* = 8; Vil/Cre, *n* = 2; and LCK/Cre, *n* = 9. Group sizes of AOM/DSS-treated mice: WT, *n* = 12; KO. *n* = 6; Vil/Cre, *n* = 4; and LCK/Cre, *n* = 8.

Although T-cells might not be the first immune response in the DSS system, they orchestrate the immune response and intestinal epithelial homeostasis. The balance between Th1 and Th17 cells is important for the fate of the tissue [22]. Therefore, we quantified the immune cell status of control and AOM/DSS-treated mice by flow cytometry. The circulating immune cells in untreated WT, CerS4 KO, CerS4 Vil/Cre, and CerS4 LCK/Cre mice were not altered. However, untreated CerS4 LCK/Cre mice had fewer CD8+ T-cells than WT mice and more CD8+ and CD4+ T-cells than KO mice (Figure 6A,B). After 12 weeks of AOM/DSS treatment, especially in CerS4 KO mice (and only by tendency in CerS4 LCK/Cre mice), DCs increased in the blood. In CerS4 LCK/Cre mice, monocytes, Tregs, and CD4+/CD8+ T-cells increased (Figure 6A). In the spleens of AOM/DSS-treated CerS4 LCK/Cre mice, levels of cDC2, NK-cells, monocytes/macrophages, and neutrophils were significantly elevated in comparison with those in the spleens of other AOM/DSS-treated mice (Figure 6B).

In conclusion, in the AOM/DSS CAC mouse, model Treg cells increased significantly in CerS4 LCK-Cre mice. This was accompanied by increases in CD4+/CD8+ T-cells and, by tendency, reduced numbers of CD4+ or CD8+ T-cells in the blood.

### 2.7. Tumors Were Enriched with Immune Cells, Particularly T-Cells, Neutrophils, and Macrophages

**Figure 7:**
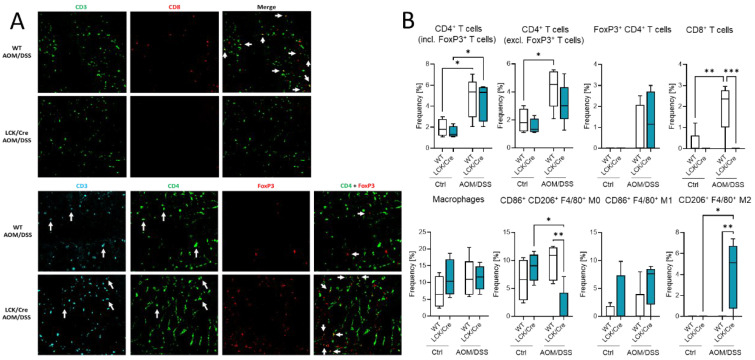

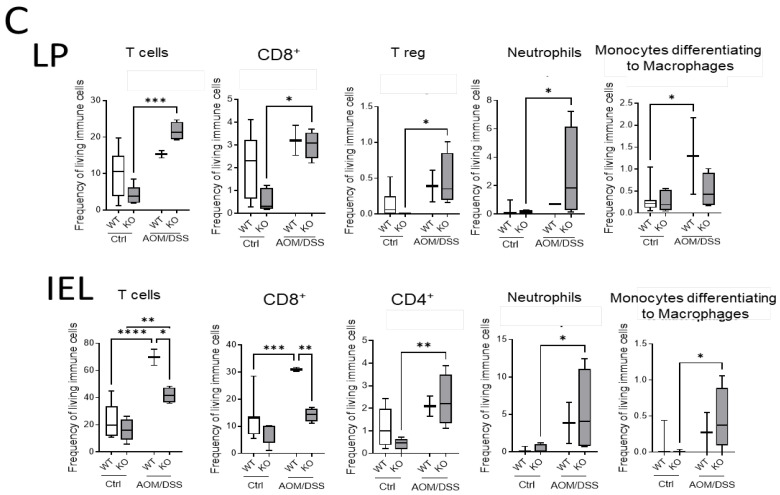
Immune cell status in colon tissue of AOM/DSS-treated mice. (**A**). MELC pictures of CD3+/CD4+ and CD3+/CD4+/FoxP3+ cells in colon tissue from AOM/DSS-treated WT and CerS4 LCK/Cre mice. (**B**) MELC-based quantification of immune cells in colon tissue from control and AOM/DSS-treated WT and CerS4 LCK/Cre mice. For each treatment, 4–5 fields of vision were analyzed. (**C**) Flow cytometry analysis of immune cells from the colons (LP and IEL) of control and AOM/DSS-treated WT and CerS4 KO mice. Upon AOM/DSS treatment, the CD8+ T-cell frequency is increased in WT and CerS4 KO mice. However, CD8+ T-cells were significantly less abundant in AOM/DSS-treated CerS4 KO mice than in WT mice. Furthermore, increased amounts of neutrophils and monocytes were detected in WT and CerS4 KO mice after AOM/DSS treatment. Median ± min to max values are plotted in box and whiskers. Statistical analysis was performed by two-way ANOVA. (* *p* < 0.05, ** *p* < 0.01, *** *p* < 0.001, **** *p* < 0.0001) Group sizes of control mice: WT, *n* = 11 and KO, *n* = 5. Group sizes of AOM/DSS-treated mice: WT, *n* = 2 and KO, *n* = 4.

The effect of different immune cell status in the blood and spleen might have also an effect on the immune presence in the colon. Histological analysis of colon tumors by multiepitope ligand cartography (MELC) exhibited a high abundance of immune cells in colon tissue of AOM/DSS-treated WT and CerS4 LCK-Cre mice (Figure 7A,B). According to our FACS data from blood, CerS4 LCK/Cre mice showed a reduced (not detectable) level of CD3+/CD8+ T cells in comparison with WT mice. We observed also significantly higher abundance of alternatively activated M2-polarized macrophages in the colons of AOM/DSS-treated CerS4 LCK/Cre mice in comparison with those of WT mice (Figure 7B).

Additionally, we quantified the immune cells in the colon by flow cytometry. TO this end, we isolated IEL and LP fractions from the colon tissue of untreated and AOM/DSS-treated CerS4 WT and CerS4 KO mice (Figure 7C). In the LP and IEL fractions of CerS4 KO control mice, the levels of T-cells, CD8+ and CD4+ T-cells, and Tregs were always lower in comparison with those in the fractions of WT mice, although not significantly. Upon AOM/DSS treatment, all these cells accumulated in these fractions along with neutrophils and monocytes that differentiated to macrophages (Figure 7C). In accordance with our data from CerS4 LCK/Cre mice, the level of CD8+ T-cells in the IEL fraction was significantly lower in AOM/DSS-treated CerS4 KO mice than in CerS4 WT mice (Figure 7C). In summary, our data demonstrated that loss of Cers4 basally altered T-cells. Additionally, macrophages, especially the protumorigenic M2 macrophages, were more abundant in the colon tissue of AOM/DSS-treated CerS4 LCK-Cre mice.

### 2.8. CerS4 Deficiency Affected T-Cell Function

#### 2.8.1. CerS4 Deficiency in Mouse Lymphocytes Impaired CD8+ T-Cell Development

**Figure 8:**
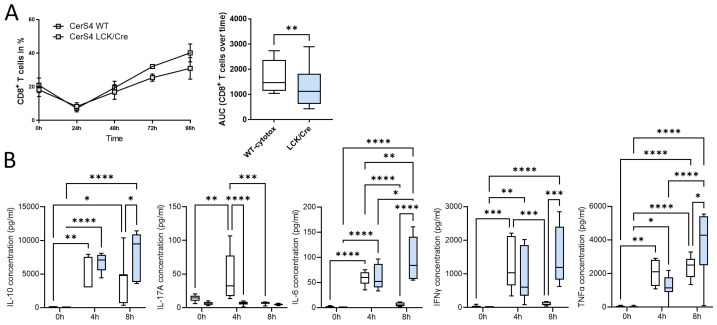
CerS4 deficiency impaired CD8+ T-cell development and enhanced cytokine secretion. (**A**) Isolated thymocytes of CerS4 WT and LCK/Cre mice were differentiated into cytotoxic T-cells. T-cells were stained with various surface markers and detected by FACS. The enrichment of CD8+ T-cells was higher in WT than in CerS4 LCK/Cre mice over the time of analysis. (**B**) For cytokine secretion, the primary T-cells were activated with IL-2 and CD2/3/28 beads for 4 h and 8 h. Cytokine concentration in the supernatant was determined with a cytometric bead array flex set (mouse IL-6, IL-10, IL-17A, IFNγ, and TNFα; BD Biosciences). Data are mean ± SEM or median ± min to max of *n* = 3–4. Statistically significant differences were determined by a two-way ANOVA with a Tukey’s multiple comparison test. (* *p* < 0.05, ** *p* < 0.01, *** *p* < 0.001, **** *p* < 0.0001).

To further investigate whether CerS4 knockdown had an impact on T-cell function, we isolated thymocytes from CerS4 LCK/Cre and WT mice and stimulated them with various cytokines to induce their differentiation into distinct T-cell subpopulations. CerS4 depletion in T-cells impaired CD8+ T-cell development (Figure 8A), but there was no influence on the development of Th1 or Tregs (data not shown). IFNγ, TNFβ, and IL-10 production was significantly reduced in cytotoxic CD8+ T-cells from CerS4 LCK/Cre cells after 4 h of stimulation. However, IL-6 production was significantly increased in comparison with that in WT cells. Interestingly, after long-term stimulation (8 h), cytokine production in WT lymphocytes decreased almost to control level, whereas in CerS4 KO T-cells, IFNγ, TNFα, IL-10, and IL-6 remained at high levels. IL-17A was significantly lower in unstimulated CerS4 KO lymphocytes than in WT lymphocytes. However, the levels were not altered after stimulation, indicating that TH17 cells were only marginally involved.

In summary, our in vitro differentiation assay revealed that depletion of CerS4 in T-cells impaired CD8+ development but simultaneously prolonged cytokine release. 

#### 2.8.2. Downregulation of CerS4 in Human Jurkat Cells Led to Constitutive Activation

**Figure 9:**
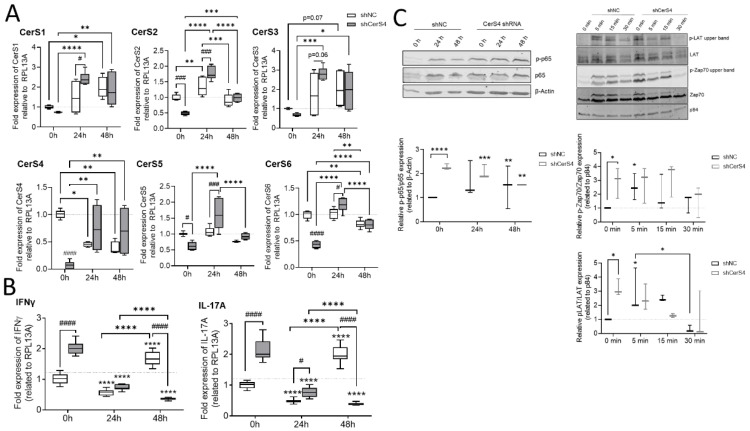
Downregulation of CerS4 in human Jurkat cells affected CerS expression and T-cell signaling. (**A**) The stimulation of transduced Jurkat cells (shNC/shCerS4) with IL-2 (200 units/mL) and anti-CD2/3/28 activation beads (1:1 bead-to-cell ratio) for 24 or 48 h changed the CerS mRNA expression profile compared with that of Jurkat shNC 0 h (set to 1). (**B**) CerS4 downregulation in Jurkat cells reduced the mRNA expression of IFNγ and IL-17a. (**C**) T-cell signaling after short- and long-term activation analyzed by Western blot. NF-κB signaling was detected by anti-phospho-p65 and anti-p65 at 0, 24, and 48 h after activation. The protein expression was normalized to β-actin. All timepoints were related to shNC Jurkat cells at 0 h (set to 1). The T-cell receptor activation was detected with anti-phospho-Zap70, anti-Zap70, phospho-LAT, and anti-LAT at short-term timepoints. Protein expression levels were normalized to p84 and compared with those of shNC Jurkat cells at 0 h (set to 1). Data are median ± min to max of *n* = 5, 3, and 2. Statistically significant differences were calculated by a two-way ANOVA with a Tukey’s multiple comparison posttest or unpaired *t*-test. (* *p* < 0.05, ** *p* < 0.01, *** *p* < 0.001, **** *p* < 0.0001).

We already showed that CerS4 was significantly downregulated in white blood cells from human colitis patients in comparison with those from control patients [12]. Therefore, we wondered how CerS4 downregulation affected T-cell function in humans. We downregulated CerS4 in human CD4+ Jurkat T-cells by shRNA treatment (shCerS4) and used a noncoding shRNA (shNC) as negative control. Interestingly, downregulation of CerS4 decreased the expression of all other CerSs significantly in unstimulated Jurkat cells (Figure 9A). After stimulation of these cells for 24 h with IL2 anti-CD3/CD28/CD2 activation beads, we observed an overcompensation in CerS4 KO cells, leading to significantly enhanced expression levels of CerS1–6 in these cells. Interestingly, in stimulated Jurkat shNC T-cells, CerS4 expression significantly decreased that after stimulation. Jurkat shNC and Jurkat shCerS4 T-cells expressed equal CerS4 levels. Therefore, downregulation of CerS4 in Jurkat T-cells mimicked the effect of long-term activation of Jurkat control T-cells by IL-2 and activation beads. This was also reflected in the IFNγ and IL-17A expression levels. Enhanced expression levels of both cytokine genes were observed in untreated Jurkat shCerS4 T-cells in comparison with Jurkat shNC T-cells. Stimulation of cells for 24 h decreased cytokine levels in both cell lines under control levels, but after 48 h, IFNγ and IL-17A mRNA levels increased in Jurkat shNC T-cells to the same level as in untreated Jurkat shCerS4 T-cells (Figure 9B). T-cell activation includes a multitude of signaling pathways [23]. Therefore, we investigated, if CerS4 influences T-cell receptor (TCR) signaling and detected phospho-LAT (Linker of activation of T-cells) and phospho-Zap 70 by Western blot analysis. In unstimulated shCers4 Jurkat T-cells, phospho-LAT and phospho-Zap 70 were significantly enhanced in comparison with unstimulated shNC Jurkat T-cells (Figure 9C). After stimulation of the TCR phospho-LAT and phospho-Zap 70 significantly increased in shNC Jurkat T-cells but not in shCerS4 Jurkat T-cells. These data indicate that downregulation of CerS4 led to a constitutively activated TCR status. 

Downstream from TCR, the transcription factor NF-κB is activated, which is an important executor of T-cell activation. To investigate whether the NF-κB pathway is also affected in CerS4 downregulated Jurkat T-cells, we detected phospho-p65 and p65 in untreated and stimulated Jurkat shNC and shCerS4 T-cells by Western blot. In untreated Jurkat shCers4 T-cells, phosphorylated NF-κB was strongly upregulated in comparison with untreated shNC T-cells (Figure 9C), indicating that this pathway was also constitutively activated. 

In conclusion, depletion of CerS4 in unstimulated Jurkat cells activated TCR signaling and mimicked long-term activation of TCR signaling. 

## 3. Discussion

We showed a tissue-specific impact of CerS4 deficiency in DSS-induced colitis and AOM/DSS-induced CAC. CerS4 KO and CerS4 LCK/Cre but not CerS4 Vil/Cre mice were more susceptible to the DSS-induced colitis and AOM/DSS-induced CAC. This was accompanied by a massive neutrophil and differentiated macrophage infiltration and inflammatory response in the colons of CerS4 KO and CerS4 LCK/Cre mice. Additionally, CerS4 KO mice suffered from high lethality in the AOM/DSS model, indicating the importance of the ubiquitously expressed CerS4 isoform. CerS4 LCK/Cre mice developed significantly more tumors per mouse in comparison with CerS4 WT, CerS4 KO, and CerS4 Vil/Cre mice and suffered from pancolitis, indicating a crucial role of CerS4 in T-cells for resolution of inflammation. 

The higher sensitivity of CerS4 KO mice to the toxic effect of AOM/DSS treatment manifested itself in a high mortality rate as early as the first days after treatment. Although the AOM dose was reduced to a double injection of 5 mg/kg, the following DSS application resulted in higher lethality in CerS4 KO (~62%) mice than in CerS4 WT (~13%) or the conditional CerS4 Cre mice (0%). AOM is metabolically activated by CYP2E1 in the liver [24] and causes dose-dependent acute toxic effects in the liver. In the long term, it induces tumors in the liver, kidneys, lungs, and large intestine [25]. Although the concentration of 20 mg/kg AOM was reported not to be hepatotoxic, AOM causes progressive liver injuries and is used as a model for investigating fulminant hepatic failure [26]. In our study, we used four times lower concentrations of AOM than in [26]. Nevertheless, CerS4 KO mice, but not CerS4 Vil/Cre or LCK/Cre mice, were highly sensitive to the toxic effect. Our LC–MS/MS data showed tremendous decreases in HexCer, Hex2Cer d18:1/18:0, dhCer d18:0/18:0, and Cer d18:1/18:1, which were accompanied by an increase in the very-long chain-ceramide C24, in the liver of untreated CerS4 KO mice in comparison with that of WT mice (Supplement Figure S1). However, no other long-chain or very-long-chain HexCer or Hex2Cer was enhanced in the liver, indicating that no other Glc/Gal- or Lac-ceramide could compensate for the loss of HexCer and Hex2Cer d18:1/18:0 in the liver. It has been shown that Cer d18:1/18:0 influences protein phosphatase 2A (PP2A) activity [27]. PP2A is involved in the transcriptional regulation of CYP2E1 and might contribute to the liver toxicity of AOM [28]. The transforming growth factor β (TGFβ) signaling pathway has also been shown to be involved in AOM-related liver toxicity [29]. The TGFβ signaling pathway is activated by CerS4 downregulation and might therefore contribute to the toxic effect of AOM [30]. However, the role of CerS4 downregulation in the liver and its effect on AOM toxicity need to be investigated in further studies and are beyond the scope of this manuscript. Interestingly, CerS2 (responsible for the production of very long chain ceramides) has already been shown to be important for normal liver function, as CerS2-KO mice suffered from hepatocarcinoma and hepatic insulin resistance [31,32].

Different studies showed that in human colon tumor tissue, expression of CerS4 and the corresponding long-chain ceramides (Cer d18:1/18.0 and Cer d18:1/20:0) decreased [17,33]. Additionally, overexpression of CerS4 in human colon cancer cells was associated with growth inhibition [34]. These data indicate that downregulation of CerS4 in colon tissue is associated with tumor-promoting effects. However, our CerS4 VilCre mouse data showed the opposite effect, which might be related to the cancer model used herein. Furthermore, our LC–MS/MS data demonstrated that long-chain ceramides did not decrease in CerS4 Vil/Cre mice. These data suggest that CerS4 downregulation in epithelial cells is not sufficient to be measurable through a decrease in long-chain ceramides in colon tissue. Therefore, the effects observed in the human colon seem unrelated to decreased expression levels in epithelial cells only. Additionally, the human data did not show if downregulation of CerS4 was the cause of colon cancer or if it occurred at later stages. Downregulation of CerS4 in early tumor stages might have opposite effects.

Beside the effect of CerS4 on liver function, CerS4 also impacted immune cell function. We observed enhanced abundance of M2-like macrophages in the colons of CerS4 LCK/Cre mice in comparison with those of WT mice after long-term treatment with AOM/DSS. M2-like macrophages polarize by interaction with Treg cells, which were also enhanced in AOM/DSS-treated CerS4 LCK/Cre mice. The high abundance of M2-like macrophages in the colon tissue of AOM/DSS-treated CerS4 LCK/Cre mice might contribute to the higher tumor incidence in these mice, because M2 cells promote tissue remodeling, angiogenesis, and tumor progression [35,36]. Additionally, Treg cells from AOM/DSS-treated mice have a suppressive effect on CD4+ and a more remarkable one on CD8+ T-cells, which was also associated with a tumor–promoting function in CAC [37]. However, in untreated CerS4 LCK/Cre mice, and by tendency in CerS4 KO mice, CD8+ T-cells were reduced in the blood and colon. In vitro, we detected a reduction in CD8+ T-cells after stimulation of lymphocytes from CerS4 LCK/Cre mice in comparison with CD8+ T-cells from WT mice. In contrast, cytokine production was prolonged in CerS4-depleted CD8+ cells after stimulation compared with CerS4-expressing CD8+ cells. IL-10 and IFNγ CD4+ cells are important for a chronic, nonresolving infection status, and together with the elevated TNFα and IL-6 expression, these data fit well with the enhanced and prolonged inflammation in the colons of AOM/DSS-treated CerS4 LCK/Cre mice [38]. Notably, TGFβ signaling has been described to inhibit T-cell proliferation by p21 induction and decreasing cyclin D1 expression but enhancing cytokine signaling after stimulation [39]. Gencer et al. showed that CerS4-produced Cer d18:1/18:0 and Cer d18:1/20:0 inhibited the TGFβ signaling pathway by stabilizing the TβRI/SMAD7 complex and that downregulation of CerS4 enhanced TGFβ signaling [30]. In CerS4-depleted CD8+ T-cells, augmented TGFβ signaling might contribute to reduced proliferation and prolonged cytokine production. Supposing a similar role of CerS4 in the TGFβ-pathway in immune cells, Garo et al., 2019 described how SMAD7 in DC and CD4 T-cells limited PD1-mediated Treg induction in colitis [40]. These results would explain why Cers4 deficiency in T-cells led to higher Treg levels in the blood and colons of AOM/DSS-treated CerS4-LCK/Cre mice. Resolution of inflammation in IBD is a promising active process to target the disease. Thiopurine or anti-tumor necrosis factor are used as immunosuppressive treatments alongside corticosteroid and antibiotic treatment. Among these approaches, anti-integrin strategies, which aim to restore the activity of TGFβ by anti SMAD7 antisense oligonucleotides [41,42,43], are also used. In IBD patients, specific antisense oligonucleotides restore TGFβ signaling and inhibit cytokine production, meaning that SMAD7 blockade of TGFβ signaling maintains chronic production of proinflammatory cytokines [44]. In an animal model, SMAD7 overexpression in CD4-T-cells prevented CAC: mice developed fewer tumors, accumulated cytotoxic CD8 cells, and had increased IFNγ levels [45]. This was in line with our study, in which CerS4 LCK/Cre mice with probably reduced SMAD7/TβRI complexes developed more tumors after AOM/DSS treatment than CerS4 WT mice. 

Besides TGFβ, the TCR signaling pathway plays an important role in tumor formation after AOM/DSS treatment [46]. In CerS4-downregulated Jurkat T-cells, the direct executors of TCR, phospho-LAT and phospho-Zap70, were only marginally affected. However, p65 was prominent and phosphorylated already in unstimulated Jurkat shCerS4 T-cells but not in unstimulated Jurkat shNC T-cells. Anti-CD28 ligation alone activates NF-κB in primary and Jurkat T-cells [47]. CerS4 downregulation leads to constitutive activation of the NF-κB signaling pathway and enhanced INFγ/IL-17 production in unstimulated Jurkat T-cells. These data let us assume that the CD28 signaling pathway was constitutively activated in Jurkat shCerS4 T-cells. CD28 is a lipid raft-associated protein [48], and downregulation of CerS4 affects the lipid raft-associated proteome (unpublished data, manuscript in preparation). Interestingly, in stimulated Jurkat shNC T-cells, CerS4 expression was significantly reduced. Therefore, downregulation of CerS4 in Jurkat T-cells via shRNA mimics CerS4 expression status in activated Jurkat T-cells. Tumor development occurs during the epithelial restoration after resolving inflammation. As TGFβ is a multifunctional cytokine, it also plays a role in colon epithelial cells. In CD or other diseases, such as chronic graft vs. host disease, overexpressing TFGβ promotes wound healing and metastasis [49]. It promotes epithelial growth and differentiation [41] and induce the secretion of defensin from Paneth cells to prevent bacterial invasion [50]. CerS4 Vil/Cre mice showed only very mild symptoms and a low number of tumors in the CAC model. These data indicate that in colon epithelial cells, TGFβ signaling may possibly play a protective role. 

Overall, our data showed that CerS4 knockdown plays an important role in colitis and CAC. Depletion of CerS4 in colon epithelial cells had a protective effect against the development of CAC. Depletion of CerS4 in T-cells inhibited resolution of inflammation after AOM/DSS treatment and enhanced tumor formation. However, in CerS4 KO mice, both mechanisms competed with each other. These mice were highly sensitive to AOM/DSS, probably because of defects in liver function. Our data shed light on the effect of CerS4 on the T-cell signaling pathway. Although CerS4 seems to be a promising drug target, strategies should be tissue specific, as CerS4 is nearly ubiquitously expressed and important for many cellular processes.

## 4. Materials and Methods

### 4.1. Animal Models

All animal experiments followed the ethical guidelines for investigations in conscious animals and the approval of the local ethics committee for animal research (FK/1082, regional council, Darmstadt, Germany). Experiments were performed with CerS4+/+ (CerS4 WT), CerS4^−/−^ (CerS4 KO), CerS4fl/fl Vil/Cre+ (CerS4 Vil/Cre), and CerS4fl/fl LCK/Cre+ (CerS4 LCK/Cre) mice. The whole CerS4 knockout (CerS4 KO) was generated by the deletion of exon 3 of the CerS4 gene by insertion of an exon 3 flanking LoxP site (CerS4 fl/fl mice) and crossing CerS4fl/fl mice with Flp-deleted Cre-deleter mice as described in [51]. We obtained those mice from Prof. Martin Krönke (Institute for Medical Microbiology, Immunology, and Hygiene (IMMIH); University of Cologne, Cologne, Germany). With the same mice, the intestinal specific CerS4 knockout mice were generated by crossing CerS4 fl/fl mice with Villin-Cre (B6.Cg-Tg(Vil1-cre)1000Gum/J), and the T-cell specific mice were generated by crossing with LCK-Cre (B6-Cg-Tg(Lck-cre)548Jxm) mice, both obtained from Jackson Laboratory (by Charles River, Sulzfeld, Germany). For all experiments, CerS4 WT had the same background and were used as control. For all experiments, male and female mice with ages of about 9 weeks were enrolled. All mice were generated in house.

The mice were housed under standard conditions (with food and water ad libitum). To analyze the role of CerS4 in inflammation and tumor development, the acute DSS model and the CAC model were applied as described in our previous studies [18,21]. For the acute DSS model, 2% (*w*/*v*) dextran sulfate sodium salt (DSS), colitis grade (36,000—50,000) (MP Biomedicals) (MP Biomedicals) was applied to drinking water for 5 days. Two to five days afterwards, the experiment was terminated, and the tissue was processed for further analysis. For the CAC model, 5 mg/kg AOM was initially injected intraperitoneally on two successive days, followed by 2% DSS application for 5 days as performed in the acute model. To induce chronic inflammation, the DSS treatment was repeated twice after a recovery of 14 days between each DSS cycle. Six weeks after the last DSS cycle, the mice were killed, and blood and tissue were collected. To determine disease progression, body weight, stool consistency, bleeding, and posture were monitored. Changes in body weight and physical status of the mice were calculated as a specific score and used to estimate an overall score for disease progression. Mice with a score >3 were excluded from the experiment. The AUC was calculated using the following formula:

(1) $$\frac{\%\;Body\;weight\cdot Time}{2}$$

### 4.2. Histological Analysis

The colon was removed, cleaned by flushing with ice cold Dulbecco’s phosphate-buffered saline 1× without calcium and magnesium (DPBS) with a syringe through the colon, and cut longitudinally. After each step, the colon was recorded with a 12× megapixel hand camera shot. Before rolling the colon from proximal to distal with two tweezers, pieces from the distal and proximal part were collected in dry ice for further analysis. The colon roll was embedded in TissueTek^®^ O.C.T.™ Compound (Sakura Finetek, Umkirch, Germany) in a Tissue-Tek^®^ Cryomold^®^ intermediate Biopsy Mold (Sakura Finetek, Umkirch, Germany), frozen on dry ice, and stored at −80 °C. Ten micrometer sections of the colon rolls were cut with LEICA CM 3050S microtome and stored for at least 24 h at −80 °C prior to staining. Colon slices (from different layers of the colon role) were stained with hematoxylin/eosin solution and observed via light microscopy with a BIOREVO (Keyence, Neu-Isenburg, Germany) microscope. Image acquisition was performed with a 10×/0.3 objective and merged with BZ II Analyzer (Keyence). The colon length was measured with FIJI software [52]. 

For immunohistochemical staining, tissue slides were fixed in 4.5% paraformaldehyde. After fixation and washing with PBS, the slices were permeabilized in PBS containing 0.025% Triton X-100 and subsequently blocked with 3% bovine serum albumin/PBS. Primary antibodies against ZO-1 (# 61-7300, Thermo Fisher Scientific, Dreieich, Germany; 1:100) or occludin (CL1608, Atlas Antibody, Sigma-Aldrich, Darmstadt, Germany; 1:150) were applied overnight at 4 °C. Fluorescence-labeled secondary antibodies, Alexa Fluor^®^ 647-anti rabbit (#A31573, Invitrogen, Dreieich, Germany; 1:1000) and Cy3-anti mouse (# C2181, Sigma, Darmstadt, Germany; 1:1000), were incubated at room temperature for 2 h. 4′,6 diamidine-2′-phenylindole dihydrochloride (DAPI, Thermo Fisher Scientific, 300 nM) was applied for 10 min at RT. After washing with PBS, the slices were mounted with PolyAquamount (Polyscience, Hirschberg, Germany). Pictures were taken with a Zeiss Axio Imager Z1 microscope with Apoptome unit.

### 4.3. Flow Cytometry

To determine the immune cell status, immune cells from blood (WBCs), spleen, and colon tissue (intraepithelial lymphocytes (IEL) and lamina propria (LP)) were isolated, and their immune cell surface markers were detected by multicolor flow cytometry. WBCs were isolated from a 1:1 mixture of EDTA blood and HEPES, lysed twice with an erythrocyte lysis buffer, washed with PBS, and resuspended in FACS flow™ (BD Bioscience, Heidelberg, Germany). For immune cell isolation from the spleen, the spleen was disrupted mechanically, and the cell suspension was washed through a 70 µm cell strainer (Corning). After erythrocyte lysis and one washing step, the cells were resuspended in FACS flow™. To isolate immune cells from the colon, IEL and LP suspensions were gained with the murine Lamina Propria Dissociation Kit (Miltenyi Biotec, Bergisch Gladbach, Germany) following the manufacturer’s instructions (descripted previously in [18]).

Single cell suspensions were blocked with FcR Blocking Reagent (Milteneyi Biotec, Bergisch Gladbach, Germany) for 15 min at room temperature, followed by 15 min staining with an antibody cocktail (CD3-PE-CF594, CD4-BV711, CD11b-BV510, CD11c-AlexaFlour700, CD14-PE, CD19-APC-H7, CD25-PE-Cy7, CD80-FITC, GITR-FITC, Ly6G-APC-Cy7, MHC-II-BV605, NK1.1-PE, 7-AAD-PE-Cy5 (BD, Heidelberg, Germany), CD45-Vioblue (Miltenyi Biotec, Bergisch Gladbach, Germany), CD8-eVolve 655, CD36-APC, Ly6C-PerCP-Cy5.5 (eBioscience, Frankfurt, Germany), and F4/80-PE-Cy7 (BioLegend, Fell, Germany)) at room temperature. After washing the single cell suspensions with DPBS (1×, gibco), cells were resuspended in FACS flow™ for flow cytometry measurement. Samples were acquired with a BD LSRFortessa™ Cell Analyzer (BD Bioscience, Heidelberg, Germany) and were analyzed using FlowJo software v10 (Treestar, Ashland, MA, USA). Gating strategy is shown in Supplementary Figure S3.

### 4.4. Transepithelial Resistance Measurment (TEER)

The human colon cancer cell line Caco-2 (ATCC HTB-37) was cultivated in MEM (+ Earle’s Salts and L-glutamine) supplemented with 10% FCS and 1% nonessential amino acids (Sigma M7145) at 37 °C in an atmosphere containing 5% CO_2_. 2 × 10^4^ Caco-2 cells were seeded in BSA-coated, transparent 1 µm 24-well ThinCert inserts (Greiner Bio-One, Frickenhausen, Germany) with 300 µL culture medium and surrounded in 1 mL culture medium in the bottom of the cellZScope2 (NanoAnalytics GmbH, Münster, Germany) for up to 14-16 days at 37 °C, 5% CO_2_. The electrical resistance was measured each hour by stimulating the cells with 1 Hz to 100 kHz, and every 3 days, the medium was replaced. In the example (Figure S2B), measurement timepoints of medium exchange were excluded to simplify the graph for the reader. The calculation of the capacity and the electrical resistance was performed by the cellZscope software (NanoAnalytics GmbH). 

### 4.5. Permeability Assay

For permeability assay, the Caco-2 cells were seeded and cultured with the same conditions as for TEER in a 24-well plate. As soon as the electrical resistance reached its maximum (after ~7 days), the transport assay was performed by adding 100 µg/mL fluorescein isothiocyanate (FITC)-dextran (average molecular weight: 40,000 by Sigma Aldrich, Darmstadt, Germany) to the upper insert. From the basolateral medium, 66.7 µL was taken at indicated timepoints up to 48 h and transferred to a black 384-well plate (Greiner Bio-One). Fluorescence was measured with the EnSpire Multimode Plate Reader (perkinElmer) at an excitation wavelength of 496 nm and an emission wavelength of 530 nm, with 100 flashes. 

### 4.6. Measurement of Sphingolipids

The quantification of sphingolipids in plasma and colon, small intestine, and liver tissue was performed by liquid chromatography tandem mass spectrometry (LC–MS/MS) as described previously [53]. In brief, the analytes were extracted by liquid–liquid extraction with methanol/chloroform/hydrochloric acid (15:83:2, *v*/*v*/*v*). The organic phase was split, evaporated to dryness, and reconstituted in methanol/formic acid (95:5, *v*/*v*) for sphingoid base measurements and tetrahydrofuran/0.2% formic acid/10 mM ammonium formate (9:1, *v*/*v*) for ceramide measurement. For both analytical runs, chromatographic separation was performed using an Aglient 1290 Infinity UHPLC system (Aglient, Waldbronn, Germany) coupled to a QTRAP 5500 tandem mass spectrometer (Sciex, Darmstadt, Germany). The analysis was conducted in Multiple Reaction Monitoring (MRM) mode. Data were acquired with Analyst Software V 1.6.3, and quantification was performed with MultiQuant Software 3.0.3 (both Sciex, Darmstadt, Germany). Reference substances for the preparation of calibration standards and quality control samples as well as internal standards were obtained from Avanti Polar Lipids (Albaster, AL, USA).

### 4.7. Cell Culture and T-cell Stimulation

The human T-cell line Jurkat (DSMZ—Deutsche Sammlung von Mikroorganismen und Zellkulturen, ACC 282) was cultured in RPMI-1640 medium with 2 mM glutamate and 10% FCS at 37 °C in an atmosphere containing 5% CO_2_. Primary T-cells isolated from the thymus were cultured in RPMI medium supplemented with 1% Pen/Strep, 1% nonessential amino acids (100×), 1% essential amino acids (MEM 50x), 1% sodium pyruvate, 1% HEPES, and 10% FCS at 37 °C (5% CO_2_) in a 24-well plate. For T-cell activation, the Jurkat cells were stimulated with 200 units/mL IL-2 (PeproTech GmbH, Hamburg, Germany) and anti-CD2/3/28 activation beads (Miltenyi Biotec, Bergisch Gladbach, Germany) in a 1:1 bead-to-cell ratio for either a long (0–48 h) or a short term (0–30 min). For harvesting, the cells were centrifuged and used either for RNA or protein extraction. 

### 4.8. CerS4 Knockdown in Human Cells

The downregulation of CerS4 in Caco-2 and Jurkat cells was performed by viral transduction with specific shRNA. In brief, vectors containing shCerS4 or a negative control (NC) shRNA with a GFP cassette were cotransfected with lentiviral packaging vectors into HEK293T cells using calcium phosphate. The viral particles were harvested with PEG-it VPS Kit (SBI System Biosciences, CA, USA) after 48 h and used for transfection of Caco-2 and Jurkat cells. Stable cell lines were selected with 1 µg/mL puromycin (InvivoGen, Toulouse, France), and transduction efficiency was monitored by fluorescence microscopy. 

### 4.9. Quantitive Real-Time PCR

To analyze any changes in CerS expression in the colon after DSS treatment, total murine RNA was isolated from 5 mg frozen colon tissue with the RNAqueous -Micro Kit^®^ (Invitrogen, Karlsruhe, Germany). The tissue was lysed with 20× volumes of lysis buffer in a swing mill (Retsch, Haan, Germany) with 3 zirconium oxide grinding balls for each sample at 25 Hz for 2.5 min. After lysis, the RNA extraction was processed per the manufacturer’s instructions, and the purified RNA was reverse transcribed to cDNA using the Verso cDNA synthesis kit (Thermo Fisher Scientific™, Waltham, MA, USA).

To quantify the CerS and cytokine expression in the Jurkat cells, mRNA was isolated with the Qiagen RNA isolation kit (Quiagen GmbH, Hilden, Germany) and eluted in 30 µL DNase/RNase free water. cDNA transcription was performed with the Verso cDNS synthesis kit (Thermo Fisher Scientific, MA, USA). 

Quantitative real-time PCR was conducted with SYBER™ Select Master Mix on the QuantStudio 5 system (both Applied Biosystems, Foster City, CA, USA). The comparative cycle threshold (CT) method was used to determine relative mRNA expression. For murine tissue, the expression level of PPIA was used to normalize CerS expression level. For Jurkat cells, the CerS level was normalized with the level of RPL17A. For detection of IL-17a and IFNγ, specific primers were used and normalized to RPL13A. The murine primers were previously described in [18], and the human CerS primer and RPL13A, in [21]. Human primer sequences for IFNγ and IL-17A were: IFNγ, 5’-TGGCATGTCAGACAGAACTTGA-3´ (forward) and 5´-TGGGTACAGTCACAGTTGTCA-3´ (reverse); IL-17A, 5’- TCCCACGAAATCCAGGATGC -3’ (forward) and 5’- GGATGTTCAGGTTGACCATCAC -3’ (reverse).

### 4.10. Cytometric Bead Array

To quantify the cytokine levels, T-cells were isolated from the thymi of Lass4 WT and Lass4 LCK/Cre mice. Primary T-cells were differentiated into cytotoxic T-cells by adding 60 ng/mL human TGFβ in the presence of IL-2 and β-mercaptoethanol. For activation, the primary T-cells were incubated with CD2/3/28-activation beads for 4 h and 8 h. The supernatant of the cell culture was harvested and stained with a cytometric bead array flex set (mouse IL-6, IL-10, IL-17A, IFNγ and TNFα; BD Biosciences). Sample acquisition was performed with a LSR Fortessa flow cytometer (BD Bioscience) and analysis was conducted with BD Biosciences FCAP software (V3.0). 

### 4.11. Western Blot

Jurkat cells were stimulated for long (0–48 h) and in short timeframes (0–30 min) with CD2/3/28 activation beads as previously described and harvested by centrifugation. Cell pellets were lysed in PhosphoSafe Extraction Reagent, supplemented with cOmplete protease inhibitor (Roche) and DMSO, and sonicated. After clearance of the protein lysate by centrifugation, protein concentration was determined with Bradford. Per Lämmli, 50 µg protein lysate was separated in a 12% sodium dodecylsulfate–polyacrylamide gel electrophoresis (SDS–PAGE) and subsequently transferred by electroblotting onto nitrocellulose membranes (Amersham Life Science, Freiburg, Germany). The membranes were blocked with 5% milk powder (in 0.1% Tween 20 in PBS supplemented with 2 mM natrium acid), and the primary antibodies were diluted in 5% milk powder (in 0.1% Tween 20 in PBS supplemented with 2 mM natrium acid) and incubated at 4 °C for 48 h using the following antibodies: phospho-NF-κB p65 (Ser536), (93H1; 1:200), NF-κB p65 (L8F6; 1:200), phospho-Zap 70 (Tyr319; Tyr352; 1:200), Zap70 (L1E5; 1:200); phosphor-LAT (Tyr171; 1:200); LAT (E3U6J; 1:200) (Cell Signaling, Leiden, Netherlands); p84 (clone 5E10 #ab487) (Abcam, Berlin, Germany); and β-actin (AC-15 #A5441 or #A2066; 1:1000) (Sigma-Aldrich, St. Louis, MO, USA). Proteins were detected by fluorescence using IRDye800- or IRDye700-conjugated secondary antibody (1:10,000; LICOR) for 35 min at RT and visualized with an Odyssey infrared scanner (LI-COR Biosciences, Bad Homburg, Germany). The protein levels were quantified with the Image Studio Lite software (LI-COR Biosciences, Bad Homburg, Germany).

### 4.12. Multiepitope Ligand Cartography (MELC)

The MELC technology is a high-throughput immunohistological imaging method that allows the visualization of 20–40 proteins on the same sample and has been described previously [54,55]. Briefly, colon tissues from AOM/DSS-treated WT and CerS4 LCK/Cre mice were embedded in tissue-freezing medium (Leica Microsystems, Nussloch, Germany). Cryosections of 10 µm thickness were applied on silane-coated coverslips, fixed in 4% paraformaldehyde in PBS for 15 min, permeabilized with 0.1% Triton X100 in PBS for 15 min, and blocked with 3% BSA in PBS for 1 h. The sample was placed on the stage of a Leica DM IRE2, and by a robotic process, the sample was incubated for 15 min with bleachable fluorescence-labeled antibodies (see Table 1).

Afterward, phase contrast and fluorescence images were taken (Apogee KX4; Apogee Instruments, Roseville, CA, USA, 2048 × 2048 pixels; final pixel size was 286 nm × 286 nm). To delete fluorescence signals, a bleaching step was performed, and the postbleaching image was recorded. Then, the next antibody was applied. For data analysis using the corresponding phase contrast images, fluorescence images produced by each antibody were aligned pixel-wise and corrected for illumination faults using flat-field correction. The postbleaching images were subtracted from their following fluorescence image.

To analyze high-dimensional MELC data, first, the illumination of all greyscale antibody channel images was corrected using CellProfiler (version 4.2.1) [56] followed by ImageJ 1.52v to diminish noise and background fluorescence and remove artifacts for further analyses if necessary. Subsequently, images for propidium iodide (cell nuclei) and CD45 were used for single-cell segmentation using CellProfiler. The resulting single-cell mask was loaded into histoCAT (version 1.76) [57] together with the corresponding antibody channel images. All images, excluding those used for single-cell mask generation, were z-score normalized and used for PhenoGraph analysis [58] as implemented in histoCAT. PhenoGraph defines phenotype clusters based on a single-cell mask and marker colocalization (k was set to 20 or 30). The resulting cell types were quantified, and the numbers of cells were given as frequencies relative to the total cell number in the field of vision.

### 4.13. Statistical Analysis and Data Presentation

Sphingolipid level, mRNA levels, MELC quantification, and flow cytometry data are presented as median ±95 percentile and min to max. Statistical analysis and graphics were performed with GraphPad Prism software (version 9.1.0; GraphPad Software, San Diego, CA). Significant differences were calculated using two-way ANOVA with Tukey’s multiple comparison posttest, one-way ANOVA with Sidak’s multiple comparison test, or two-tailed *t*-test. For time courses for disease progression and electrical capacity, area under the curve (AUC) values were calculated by GraphPad Prism, and statistically significant differences were analyzed by two-tailed *t*-test. 

## Figures and Tables

**Table 1:** Antibody list used for MELC analysis (Figure 7).

Name	Label	Host	Company	Clone	Cat. Number
CD4	FITC	rat	SoutherBiotech	L3T4	1540-02
CD3	APC		Miltenyi Biotec	REAG1	130-109-838
CD8a	FITC	rat	BDPharmingen	53-6.7	553030
CD11b	FITC	rat	BioRad	M1/70.15	MCA74F
CD45	FITC	rat	Biolegend	30F11	103108
CD86	PE	rat	Biolegend	GL-1	105008
CD206	APC	rat	Biolegend	C068C2	141708
F4-80	PE	rat	Biolegend	BM8	123110
FoxP3	PE	rat	eBioscience	FJK-16s	12-5773-82
Propidiumiodide			Sigma Aldrich		P4170

## Data Availability

Not applicable.

## Supplementary Materials

The following supporting information can be downloaded at: www.mdpi.com/article/10.3390/ijms23031866/s1.
