# Supplementary material for: T-Cell-Specific CerS4 Depletion Prolonged Inflammation and Enhanced Tumor Burden in the AOM/DSS-Induced CAC Model
Source: Int J Mol Sci. 2022 Feb 7;23(3):1866. doi: 10.3390/ijms23031866 (PMC8837088; doi:10.3390/ijms23031866)
Supplement: Supplementary file 1 [file ijms-23-01866-s001.zip › ijms-1533584-supplementary.pdf]

Table S1.

Data to Figure 4A colon.

	WT Ctrl			KO Ctrl			WT DSS			KO DSS		
	Mean	SEM	N	Mean	SEM	N	Mean	SEM	N	Mean	SEM	N
SPH d18:1	242.875	65.1799736	4	314.3	47.5421567	4	752.3125	158.463748	16	726.654444	98.9466393	18
SPH d18:0	524.15	126.934901	4	537.85	136.214014	4	435.9625	57.1368087	16	485.138889	67.7825142	18
Cer d18:0/16:0	2516.4	625.549516	4	4082.3	1819.13941	4	2029.18125	223.514129	16	2585.95556	355.768728	18
Cer d18:0/18:0	144.425	14.5468424	4	108.675	17.2134335	4	211.3125	23.1955705	16	**123.517647**	8.97083606	17
Cer d18:0/24:0	166.5	0	1	180.75	15.65	2	214.95	22.385153	12	287.64	35.2244145	10
Cer d18:0/24:1	203.9	0	1	202.3	45.5	2	231.3	23.7462039	14	328.713333	59.1659554	15
Cer d18:1/14:0	219.775	30.1139053	4	266.025	91.6917697	4	167.425	29.4870996	16	154.244444	21.5913203	18
Cer d18:1/16:0	38387.5	6371.70846	4	**47257.5**	12042.9818	4	**64181.3794**	10915.3837	16	59930.4467	8818.51851	18
Cer d18:1/18:0	4227.075	1097.0572	4	3637.475	1317.277	4	**7349.49375**	961.598559	16	6156.52222	795.497182	18
Cer d18:1/18:1	553	89.6570596	4	646.066667	43.1836903	3	714.728571	63.3554697	14	662.9	62.4412042	18
Cer d18:1/20:0	3236.425	676.206093	4	998.3	333.010558	4	**6739.65**	866.057628	16	**1921.54444**	203.724018	18
Cer d18:1/22:0	2899.3	587.999529	4	2984.675	677.660639	4	5076.04375	443.581306	16	4894.88333	406.588887	18
Cer d18:1/24:0	2653.625	467.21608	4	2999.85	604.564704	4	3887.96875	242.755232	16	4449.31667	266.806404	18
Cer d18:1/24:1	8219.225	1498.90266	4	8395.275	1269.89398	4	11151.4375	796.715922	16	**13033.4611**	936.120639	18
HexCer d18:1/16:0	3480.7	617.460667	4	3477.775	533.003501	4	7900.30625	902.91845	16	10717.7647	1583.79974	17
HexCer d18:1/18:0	1003.8	218.899029	4	516	101.874907	4	2520.20938	387.883412	16	1267.97222	135.192711	18
HexCer d18:1/24:1	1619.93333	45.3799638	3	1720.5	26.1996819	3	**6027.10625**	739.569313	16	**5875.80556**	854.891403	18
Hex2Cer d18:1/18:0	93.35	2.55	2	110.8	0	1	176.378571	15.9404702	14	104.728571	9.31150434	14
Hex2Cer d18:1/24:0	312.9	10.3	2	329.366667	12.9236648	3	721.93125	104.724331	16	584.86875	52.3339897	16

Data to Figure 4B small intestine

	WT Ctrl			KO Ctrl			WT DSS			KO DSS		
	Mean	SEM	N	Mean	SEM	N	Mean	SEM	N	Mean	SEM	N
SPH d18:1	1011.81	617.554983	4	1030.30429	206.668845	7	918.35	99.8290731	6	1035.6	197.116108	7
SPH d18:0	642.5975	159.352753	4	636.574286	77.3518268	7	546.133333	177.978352	6	460.471429	118.534284	7
Cer d18:0/16:0	7969.4	1597.6851	3	**3393.7**	754.916653	7	4958.01667	418.173092	6	10590.5333	4595.6715	6
Cer d18:0/18:0	181.033333	12.731108	3	114.3	26.0743488	4	171.383333	5.72988753	6	211.316667	37.1940086	6
Cer d18:0/24:0	660.433333	156.855999	3	600	76.1347865	7	518.016667	42.211463	6	1938.81429	644.977634	7
Cer d18:0/24:1	685.233333	58.2254335	3	560.442857	118.826201	7	586.866667	51.1608618	6	1446.07143	516.342104	7
Cer d18:1/14:0	400.5	153.325927	3	334	53.1057794	7	232.95	15.7974629	6	184.457143	31.9813328	7
Cer d18:1/16:0	64286.4667	25205.2892	3	69691.8714	16446.4863	7	79460	13925	2	**123835**	35365	2
Cer d18:1/18:0	7192.73333	3338.7895	3	**4314.68571**	473.759382	7	6128.63333	728.187932	6	**3120.58571**	404.302776	7
Cer d18:1/18:1	799.8	10	2	816.85	97.95	2	907.516667	26.8566245	6	783.085714	90.141118	7
Cer d18:1/20:0	3528.1	1021.4022	3	905.185714	103.770662	7	6289.06667	772.660841	6	**1108.85714**	173.60893	7

Cer d18:1/22:0	6214.66667	746.160987	3	5334.22857	578.00415	7	5419.73333	421.027907	6	**4663.21429**	577.883865	7
Cer d18:1/24:0	6962.33333	1050.68781	3	5429.48571	416.946622	7	4170.1	251.956826	6	4548.32857	410.176424	7
Cer d18:1/24:1	13979.9333	4362.61875	3	12989.1429	1068.87893	7	11160	712.877892	6	**9258.77143**	1067.13697	7
HexCer d18:1/16:0	9934.5	2205.13792	3	8865.51429	1338.80418	7	**4847.53333**	854.921916	6	**5506.08571**	1092.65953	7
HexCer d18:1/18:0	1098.9	45.4495691	3	721.1	68.3122941	7	849.916667	133.017892	6	394.685714	53.7804639	7
HexCer d18:1/24:1	2442.6	583.943528	3	3497.62857	389.166417	7	2460.85	686.276416	6	1306.71429	289.388376	7
Hex2Cer d18:1/18:0	282.5	0	1	154.06	54.0759152	5	82.82	6.42140172	5	62.0666667	6.6972963	6
Hex2Cer d18:1/24:0	306.3	5.8	2	397.575	16.2059338	4	299.466667	33.888933	6	338.228571	53.9319753	7

Data to Figure 4c liver

	WT Ctrl			KO Ctrl			WT DSS			KO DSS		
	Mean	SEM	N	Mean	SEM	N	Mean	SEM	N	Mean	SEM	N
SPH d18:1	376.6375	136.9780387	4	534.49	172.6992682	7	1186.026667	224.9227432	6	1207.598571	253.2255356	7
SPH d18:0	464.9775	170.7644577	4	494.536	161.1479231	5	681.5833333	206.3291956	6	537.2	124.3765749	7
Cer d18:0/16:0	462.55	194.2952586	4	239.1428571	25.83898731	7	250.0333333	30.41622009	6	237.7285714	34.76865525	7
Cer d18:0/18:0	206.3	102.5	2	279.4	157.5631408	3	137.6	32.71342742	4	82.3	0	1
Cer d18:0/24:0	1770.475	1403.941572	4	5528.642857	3383.298108	7	299.6333333	34.77779624	6	275.1714286	31.72922428	7
Cer d18:0/24:1	3856	2926.559345	4	5596.128571	3128.344082	7	542.6166667	60.17508575	6	348.6857143	48.46239302	7
Cer d18:1/16:0	5465.75	1464.350858	4	4816.828571	647.8570616	7	4381.383333	646.2860674	6	4926.271429	590.8574164	7
Cer d18:1/18:0	1040.35	244.4992076	4	1311.614286	880.9834773	7	814.1666667	157.5926493	6	590.6	128.8945749	7
Cer d18:1/20:0	3520.25	2421.650343	4	3426.871429	1208.299749	7	3145.783333	652.6432044	6	1968.314286	388.3101328	7
Cer d18:1/22:0	6486.366667	241.0046081	3	**17364.52**	6420.89105	5	13312.435	4038.382961	6	**9695.1**	1995.542233	6
Cer d18:1/24:0	10996.8	3694.773437	4	13095.68333	2666.113822	6	13739.16667	1719.187999	6	**19795.04286**	1920.210689	7
Cer d18:1/24:1	26694.13333	1200.467346	3	27978.68	2816.322575	5	22856.33333	864.5276532	6	**22727.42857**	2464.06364	7
HexCer d18:1/16:0	7956.425	2688.218373	4	10545.5	2103.330359	5	**13437.45**	3553.708019	6	10003.85714	1613.152927	7
HexCer d18:1/18:0	1574.85	377.8926843	4	871.8	265.1423071	5	1745.118333	610.5847676	6	1425.788571	795.8566729	7
HexCer d18:1/24:1	8874.033333	177.147644	3	10126.5	1100.427126	5	12034.63333	1042.82367	6	9789.871429	1078.160247	7
Hex2Cer d18:1/18:0	142.6	0	1	97.45	32.25	2	57.85	17.41046141	4	33.4	3.611094017	5
Hex2Cer d18:1/24:0	353	9.7	2	405.02	24.34116678	5	621.6	78.89764255	6	632.1285714	82.27254855	7

Data to Figure 4D plasma

	WT Ctrl			KO Ctrl			WT DSS			KO DSS		
	Mean	SEM	N	Mean	SEM	N	Mean	SEM	N	Mean	SEM	N
SPH d18:0	6.405	1.114783238	4	10.08571429	5.019851476	7	14.08166667	3.567164529	6	12.795	3.085807026	6
S1P d18:1	313.295	177.3018634	4	464.6971429	116.2450129	7	67.64333333	53.960062	6	**10.45833333**	2.263964689	6
S1P d18:0	202.2175	18.74960061	4	239.35	19.42364735	7	289.7616667	50.33710318	6	358.9733333	63.12461568	6
Cer d18:0/16:0	498.585	76.485	2	253.3175	140.9080228	4	291.344	89.70729327	5	241.5866667	63.75283631	6
Cer d18:0/18:0	12.155	3.475	2	14.3	4.93	2	10.53	0	1	9.99	0.54	2
Cer d18:0/24:1	26.71	0	1	36.43	9.501948923	3	23.345	3.025	2	25.915	1.115	2
Cer d18:1/16:0	32.96666667	7.406983942	3	73.7625	21.36025803	4	25.036	6.1153811	5	20.238	5.612874843	5

Cer d18:1/22:0	67.04	48.15	2	352.834	200.7425517	5	148.85	89.9286836	3	34.955	9.535	2
Cer d18:1/24:0	118.755	32.20987879	4	330.0442857	102.411743	7	255.05	97.40811284	6	170.79	76.52077456	5
Cer d18:1/24:1	262.31	52.41612554	4	378.28	86.16592742	7	300.82	53.18380007	5	469.9766667	116.1752339	3
HexCer d18:1/16:0	476.7725	223.0027574	4	471.3483333	82.71982291	6	465.198	158.2154086	5	262.34	61.18762157	3
HexCer d18:1/18:0	680.0225	269.8669429	4	276.9142857	90.0420214	7	650.1316667	281.4637188	6	**760.31**	324.6837052	6
HexCer d18:1/18:1	111.63	23.0521127	3	44.502	22.77273576	5	45.174	15.68798795	5	50.07166667	20.70511071	6
HexCer d18:1/24:1	282.46	275.6851862	3	457.3525	152.4621096	4	253.55	245.7845947	5	6.59	2.638136969	4
Hex2Cer d18:1/16:0	895.2733333	324.4670561	3	612.005	152.1130842	4	741.156	199.1638862	5	606.7516667	202.769575	6

Table S2.

Data to Supplement Figure S1A colon									
	WT Ctrl			KO Ctrl			Vil/Cre Ctrl		
	Mean	SEM	N	Mean	SEM	N	Mean	SEM	N
SPH d18:1	982.4908333	80.92693538	24	1042.668333	95.06270837	6	1026.2	205.5008881	4
SPH d18:0	161.06	25.15485297	22	284.0983333	42.17699028	6	101.725	12.45775361	4
S1P d18:1									
S1P d18:0	852.5	0	1	28.88	0	1			
Cer d18:0/16:0	1398.877778	332.1478025	9	1784.9	267.5652506	6			
Cer d18:0/18:0	555.9722222	56.39590376	18	90.96666667	8.584896298	6	762.5	126.0769144	4
Cer d18:0/24:0	187.7	24.7	2	167.05	9.75	2			
Cer d18:0/24:1	4161.495833	700.0447877	24	247.1	33.89184268	5	6274.25	1064.507101	4
Cer d18:1/14:0	274.2625	91.87435508	8	492.7333333	114.3455892	6			
Cer d18:1/16:0	11963.44583	3484.361696	24	54847	11733.27442	4	2830.25	307.6811025	4
Cer d18:1/18:0	3564.245833	366.5303709	24	**5861.233333**	1381.326399	6	2461.15	257.2895046	4
Cer d18:1/18:1	2901.3125	251.5767687	16	**272.55**	0.35	2	2185.525	231.1970638	4
Cer d18:1/20:0	7820.325	896.5416106	24	1020.833333	318.5148407	6	9674	1097.361895	4
Cer d18:1/22:0	3769.3875	310.434451	8	3682.283333	533.431441	6			
Cer d18:1/24:0	4055.333333	406.7821629	24	3342.033333	216.1832289	6	2904.175	282.0266931	4
Cer d18:1/24:1	4760.608333	986.378067	24	**11832**	1037.034588	6	1167.625	179.5562325	4
HexCer d18:1/16:0	2079.8	347.6084483	7	3372.2	527.4823864	6			
HexCer d18:1/18:0	2605.554167	414.3297437	24	622.3	84.29686827	6	1706.65	114.442187	4
HexCer d18:1/18:1	672.9666667	55.0846722	3	62.3	1.3	2			
HexCer d18:1/24:1	2317.7125	160.4348938	8	2780.233333	441.5686414	6			
Hex2Cer d18:1/16:0	684.7142857	84.13720775	7						
Hex2Cer d18:1/18:0	529.83	97.96264812	20	180.4	31.25873745	6	394.6666667	47.54230864	3
Hex2Cer d18:1/24:0	1022.558333	269.1798755	12	343.7	16.1	2			

Hex2Cer d18:1/24:1	1020.3	128.1	2	753.4	26.26505917	3

	WT AOM/DSS			KO AOM/DSS			Vil/Cre AOM/DSS		
	Mean	SEM	N	Mean	SEM	N	Mean	SEM	N
SPH d18:1	1095.472727	57.47947655	22	1009.1	162.1785288	6	1186.185714	121.8719535	7
SPH d18:0	213.1363636	23.43279378	22	274.8916667	80.94971708	6	137.9285714	12.87649602	7
S1P d18:1									
S1P d18:0				71.15	0	1	819.7	0	1
Cer d18:0/16:0	1825.188889	223.9886188	18	1840.233333	502.4517255	6	382.1	0	1
Cer d18:0/18:0	264.6571429	48.80596333	14	142	27.8192679	4	633.2285714	56.66195379	7
Cer d18:0/24:0	297.2833333	32.97888634	6	292.2	88.5	2			
Cer d18:0/24:1	1359.773684	473.4271663	19	301.3166667	58.55129043	6	7970.057143	1327.821605	7
Cer d18:1/14:0	172.8444444	15.23382722	18	151.9333333	40.61505194	6			
Cer d18:1/16:0	**20832.28571**	3355.653029	14	**21655.675**	8385.078494	4	3348.471429	269.9457871	7
Cer d18:1/18:0	4979.25	610.0833678	22	4553.316667	1412.46899	6	3437.814286	306.3703147	7
Cer d18:1/18:1	999.15	328.0688443	12	274	10.5	2	3992.428571	618.4299817	7
Cer d18:1/20:0	6291.177273	615.4004164	22	1455.9	403.793571	6	12229.94286	945.8739629	7
Cer d18:1/22:0	4946.883333	318.5334438	18	3975.783333	794.6879251	6			
Cer d18:1/24:0	4156.586364	272.1128241	22	3786.266667	523.0159288	6	5289.085714	421.6499301	7
Cer d18:1/24:1	**11390.05909**	1224.714496	22	12320	1727.95754	6	2024.271429	109.9649743	7
HexCer d18:1/16:0	6431.255556	1355.753683	18	8973.083333	3688.236831	6			
HexCer d18:1/18:0	2085.446667	394.1092336	15	1366.766667	246.4817636	6	3986.757143	466.3546892	7
HexCer d18:1/18:1	186.72	87.99582288	10	55.25	3.45	2	580.9	0	1
HexCer d18:1/24:1	6390.427778	1015.810478	18	6707.566667	1992.36667	6			
Hex2Cer d18:1/16:0	2137.457143	439.2103914	14	1187	183.9004124	4			
Hex2Cer d18:1/18:0	275.6681818	46.13421901	22	221.1833333	71.78255785	6	810.0285714	86.87468729	7
Hex2Cer d18:1/24:0	750.905	101.6923597	20	636.6166667	113.9180039	6	1590.55	143.3200876	4
Hex2Cer d18:1/24:1	1490.671429	115.0237692	7	2055.65	147.45	2			

Data to Supplement Figure S1A liver

	WT Ctrl			KO Ctrl			Vil/Cre Ctrl		
	Mean	SEM	N	Mean	SEM	N	Mean	SEM	N
SPH d18:1	1004.5025	115.2686749	12	1011.043333	63.40709223	3	1402.85	285.68	2
SPH d18:0	216.015	21.81526872	12	165.2666667	24.66533217	3	338.4	78.49	2
S1P d18:1	21.87	8.926539083	3						
S1P d18:0	17.83333333	5.12283884	3						
Cer d18:0/16:0	223.6	0	1	149	24.1508454	3			
Cer d18:0/18:0	82.2	0	1						
Cer d18:0/24:0	182.0555556	24.90709466	9	170	10.5	2	128.2	0.6	2
Cer d18:0/24:1	296.8142857	65.086124	7	196.9	25.14597383	3			

Cer d18:1/14:0	186.3714286	51.60930478	7	22.4	0.2	2	182.1	4.3	2
Cer d18:1/16:0	1490.966667	380.33791	6	4119.9	655.2996338	3	391.2	56.2	2
Cer d18:1/18:0	268.15	105.3290332	12	311.6666667	86.65966638	3	79.7	1.3	2
Cer d18:1/18:1	6047.5	571.4625141	8				5232.25	378.25	2
Cer d18:1/20:0	1166.85	199.4254021	12	**749.8**	48.48095846	3	861.7	98.9	2
Cer d18:1/22:0	3836.641667	1745.62908	12	14206.56667	4528.245044	3	470.15	59.75	2
Cer d18:1/24:0	11788.2	1206.735881	4	16701.33333	1020.408291	3			
Cer d18:1/24:1	7474.708333	2779.161994	12	**20855.93333**	2882.460911	3	716.85	56.35	2
HexCer d18:1/16:0	9719.75	1255.605081	12	4725.333333	532.3095006	3	14487.8	597.9	2
HexCer d18:1/18:0	16578.8	3170.130166	11	459	227.7420763	3	25471.25	320.15	2
HexCer d18:1/18:1	9367.555556	1593.038593	9				8805.8	1273.5	2
HexCer d18:1/24:1	5976.075	1555.586101	12	7234.1	1214.89882	3	1287.55	279.35	2
Hex2Cer d18:1/16:0	402.4	121.8506531	4						
Hex2Cer d18:1/18:0	8908.190909	**1831.225586**	11	48.15	22.45	2	11748.45	2514.55	2
Hex2Cer d18:1/24:0	340.75	103.1274091	4	459.6	0	1			
Hex2Cer d18:1/24:1	894.5	0	1	915.8	0	1			

	WT AOM/DSS			KO AOM/DSS			Vil/Cre AOM/DSS		
	Mean	SEM	N	Mean	SEM	N	Mean	SEM	N
SPH d18:1	1241.82	191.7040672	5	1708.646667	318.7093799	3	370.665	63.39390737	4
SPH d18:0	355.5666667	50.12555347	9	661.1333333	242.2710076	3	97.3275	20.86909572	4
S1P d18:1							29.48333333	12.05897499	3
S1P d18:0							16.725	3.655	2
Cer d18:0/16:0	207.5888889	16.4269464	9	365.2666667	162.5335487	3			
Cer d18:0/18:0	76.2	0	1	262.5	0	1			
Cer d18:0/24:0	255.35	19.29855349	4	295.65	55.05	2	144.025	14.872871	4
Cer d18:0/24:1	494.8777778	71.02692326	9	642.7	115.7636529	3	93.1	10.78162016	3
Cer d18:1/14:0	17	0	1	79.9	0	1	290.7	35.11248591	4
Cer d18:1/16:0	2964.244444	225.437529	9	3429.533333	496.6423607	3	761.125	70.24811237	4
Cer d18:1/18:0	416.3888889	78.36024439	9	1213.433333	712.9355846	3	105.65	6.121614711	4
Cer d18:1/18:1							7103.825	381.1548381	4
Cer d18:1/20:0	**1330.788889**	217.6710167	9	1155.166667	417.6415302	3	1313.675	177.3357756	4
Cer d18:1/22:0	9768.511111	1239.801008	9	10341.33333	5387.070413	3	551.95	14.86250876	4
Cer d18:1/24:0	12566.6	1191.099151	9	15530.43333	3977.097831	3			
Cer d18:1/24:1	**26741.7**	2110.448822	9	**30974.66667**	7548.0657	3	851.175	74.76125751	4
HexCer d18:1/16:0	6102.444444	987.5357438	9	9211.1	2455.287507	3	13797.2	983.9459055	4
HexCer d18:1/18:0	638.325	141.4811317	8	1334.8	155.3286945	3	24830.875	688.8666337	4
HexCer d18:1/18:1	20.35	0.35	2	18.2	0	1	12918.2	1653.800126	4
HexCer d18:1/24:1	16654.55556	3111.830853	9	13848.06667	3247.988205	3	2734.5	503.7341528	4

Hex2Cer d18:1/16:0	786.82	80.62285904	5	797.35	138.95	2			
Hex2Cer d18:1/18:0	**46.95**	6.314241273	8	74.5	38.71317605	3	13223.75	1154.513452	4
Hex2Cer d18:1/24:0	384.6777778	30.5368756	9	612.3333333	158.0015014	3			
Hex2Cer d18:1/24:1	1427.8	0	1	1705.2	0	1			

Data to Supplement Figure S1A plasma

	WT Ctrl			KO Ctrl			Vil/Cre Ctrl		
	Mean	SEM	N	Mean	SEM	N	Mean	SEM	N
SPH d18:1	9.228	0.837128425	5	15.84	2.54	2			
SPH d18:0	7.496	0.859619683	5	6.803333333	1.556516767	3			
S1P d18:1	428.376	101.6279627	5	180.18	142.8453188	3			
S1P d18:0	146.875	26.96155012	6	203.9433333	21.16728162	3			
Cer d18:0/16:0				10.83	0	1			
Cer d18:0/18:0									
Cer d18:0/24:0	39.987	9.454510041	10	30.555	12.935	2	35.155	11.995	2
Cer d18:0/24:1	17.81666667	2.375502286	3						
Cer d18:1/14:0									
Cer d18:1/16:0	35.18285714	5.715815033	7	81.345	31.095	2			
Cer d18:1/18:0	152.8771429	66.70885815	7				38.55	11.08	2
Cer d18:1/18:1	220.55	54.83528554	6				96.03	17.37	2
Cer d18:1/20:0	184.97	59.40358953	10	309.9	0	1	142.015	27.615	2
Cer d18:1/22:0	496.928	117.3008137	5	4725.1	2600.9	2			
Cer d18:1/24:0	540.2783333	75.18967095	12	**5432.15**	2666.65	2	337.085	30.355	2
Cer d18:1/24:1	307.9269231	90.26157515	13	**3562.55**	949.45	2	44.71	2.46	2
HexCer d18:1/16:0	820.3383333	540.2281883	6	1603.133333	701.9149529	3			
HexCer d18:1/18:0	548.0538462	138.9584724	13	121.7133333	50.22881156	3	462.84	104.18	2
HexCer d18:1/18:1	4.145	1.005	2	2.74	0	1			
HexCer d18:1/24:1	1790.088	723.9842406	5	4542.843333	2210.390655	3			
Hex2Cer d18:1/16:0	135.745	6.315	2						

	WT AOM/DSS			KO AOM/DSS			Vil/Cre AOM/DSS		
	Mean	SEM	N	Mean	SEM	N	Mean	SEM	N
SPH d18:1	9.487777778	1.403085665	9	17.80333333	12.77882928	3			
SPH d18:0	9.872222222	1.77267906	9	15.55333333	10.36603642	3			
S1P d18:1	533.6511111	35.82560062	9	343.41	182.832324	3			
S1P d18:0	235.2866667	25.78277935	9	259.26	38.99881579	3			
Cer d18:0/16:0				22.8	0	1			
Cer d18:0/18:0				11.17	0	1			
Cer d18:0/24:0	48.945	40.525	2	103.93	0	1	26.8975	23.18384836	4
Cer d18:0/24:1				161.54	0	1			

Cer d18:1/14:0									
Cer d18:1/16:0	25.85	9.482789674	3	201.44	0	1			
Cer d18:1/18:0	174.375	158.605	2	250.8	0	1	32.84	21.74886817	4
Cer d18:1/18:1	400.82	0	1				113.06	34.3795317	2
Cer d18:1/20:0	195.57	129.5107204	3	483.7	0	1	160.885	76.41702984	2
Cer d18:1/22:0	120.6585714	63.5460296	7	2472.495	2450.605	2			
Cer d18:1/24:0	333.4875	68.9249347	4	**9566.6**	0	1	376.0875	179.6780835	4
Cer d18:1/24:1	194.014	88.95677122	5	**9883.8**	0	1	88.905	49.37394353	4
HexCer d18:1/16:0	236.2171429	35.82895389	7	**5091.216667**	4906.180995	3			
HexCer d18:1/18:0	147.4418182	101.8035624	11	722.7233333	691.9902419	3	436.1375	217.6687536	4
HexCer d18:1/18:1	2.32	0.212994523	4	2.265	0.175	2			
HexCer d18:1/24:1	**330.6788889**	79.35481048	9	5192.423333	4970.706813	3			
Hex2Cer d18:1/16:0									

Data to Supplement Figure S1B plasma

	WT Ctrl			KO Ctrl			LCK/Cre Ctrl		
	Mean	SEM	N	Mean	SEM	N	Mean	SEM	N
SPH d18:1	9.1255	2.3845	2				10.189	1.451	2
SPH d18:0	6.344166667	0.788308756	6	10.08571429	5.019851476	7	7.073	1.328	2
S1P d18:1	419.0468333	130.8411167	6	464.6971429	116.2450129	7	705.3385	127.0455	2
S1P d18:0	191.2391667	13.84829835	6	239.35	19.42364735	7	216.629	45.63	2
Cer d18:0/16:0	498.585	76.485	2	253.3175	140.9080228	4			
Cer d18:0/18:0	12.155	3.475	2	14.3	4.93	2			
Cer d18:0/24:0	15.87	0	1	24.97333333	9.769969862	3			
Cer d18:0/24:1				36.43	9.501948923	3	16.06	0	1
Cer d18:1/14:0	26.71	0	1	1.83	0	1			
Cer d18:1/16:0	37.12	10.21	2	49.5175	22.60154581	4			
Cer d18:1/18:0	44.11	0	1	73.7625	21.36025803	4	48.135	21.225	2
Cer d18:1/18:1	32.96666667	7.406983942	3	20.49	0	1	7.5	0	1
Cer d18:1/20:0				52.14	18.8	2			
Cer d18:1/22:0	70.92	9.24	2	352.834	200.7425517	5	30.17	0	1
Cer d18:1/24:0	125.6925	51.50287377	4	330.0442857	102.411743	7	245.45	183.77	2
Cer d18:1/24:1	125.7816667	22.58079219	6	378.28	86.16592742	7	253.595	151.205	2
HexCer d18:1/16:0	262.31	52.41612554	4	471.3483333	82.71982291	6	252.86	146.6	2
HexCer d18:1/18:0	324.9033333	170.6594587	6	276.9142857	90.0420214	7			
HexCer d18:1/18:1	680.0225	269.8669429	4	**44.502**	22.77273576	5	**30.06**	1.25	2
HexCer d18:1/24:1	196.058	53.75564439	5	457.3525	152.4621096	4			
Hex2Cer d18:1/16:0	282.46	275.6851862	3	612.005	152.1130842	4	506.82	160.2	2

	WT DSS			KO DSS			LCK/Cre DSS		

	Mean	SEM	N	Mean	SEM	N	Mean	SEM	N
SPH d18:1	11.9538	2.359197245	5				16.2976	5.488327292	5
SPH d18:0	11.79163636	2.154956799	11	**12.795**	3.085807026	6	**9.0984**	2.057269102	5
S1P d18:1	294.7423636	84.21526782	11	10.45833333	2.263964689	6	615.4402	56.36268799	5
S1P d18:0	219.352	36.24558465	11	358.9733333	63.12461568	6	141.7636	11.02129735	5
Cer d18:0/16:0	247.8583333	85.18177925	6	241.5866667	63.75283631	6	30.43	0	1
Cer d18:0/18:0	10.53	0	1	9.99	0.54	2			
Cer d18:0/24:0	17.91	0	1						
Cer d18:0/24:1	37.6075	13.36575561	4	25.915	1.115	2	39.936	10.39317305	5
Cer d18:1/14:0				16.2	0	1			
Cer d18:1/16:0	47.18	0	1						
Cer d18:1/18:0	37.724	11.41791363	10	20.238	5.612874843	5	74.034	19.84997673	5
Cer d18:1/18:1	8.5	0	1						
Cer d18:1/20:0	58.62	0	1						
Cer d18:1/22:0	114.25	72.39298355	4	34.955	9.535	2	8.82	0	1
Cer d18:1/24:0	175.6363636	58.60993585	11	170.79	76.52077456	5	128.056	36.03763472	5
Cer d18:1/24:1	270.044	54.59641206	10	469.9766667	116.1752339	3	408.702	104.4145235	5
HexCer d18:1/16:0	315.7	91.03465039	10	262.34	61.18762157	3	287.748	88.34973398	5
HexCer d18:1/18:0	535.523	169.6098106	10	**760.31**	324.6837052	6	496.235	35.135	2
HexCer d18:1/18:1	**38.998**	8.277058629	10	50.07166667	20.70511071	6	20.94	3.475666842	5
HexCer d18:1/24:1	253.55	245.7845947	5	6.59	2.638136969	4			
Hex2Cer d18:1/16:0	620.681	107.7010749	10	606.7516667	202.769575	6	542.408	86.55766375	5

Data to Supplement Figure S1B thymus

	WT Ctrl			LCK/Cre Ctrl			WT DSS			LCK/Cre DSS		
	Mean	SEM	N	Mean	SEM	N	Mean	SEM	N	Mean	SEM	N
SPH d18:1	311.945	43.495	2	388.51	33.4894615	3	465.494	120.893833	5	563.84	162.325375	5
SPH d18:0	63.09	10.7	2	105.053333	22.5422162	3	96.604	27.9856816	5	141.162	55.2143358	5
S1P d18:1							63.33	0	1	73.875	26.225	2
S1P d18:0							33.835	9.615	2	34.08	6.6	2
Cer d18:0/16:0	853.8	89.3	2	681.066667	184.141362	3	677.4	111.974939	5	787.18	242.208362	5
Cer d18:0/18:0	74.7	13.1	2	111.4	0	1	117.125	39.1120479	4	133.166667	31.5944264	3
Cer d18:0/24:0				183.1	0	1	313	131.7	2	316.4	12.1	2
Cer d18:0/24:1				650.4	0	1	607.6	0	1	850.8	0	1
Cer d18:1/14:0	103.35	7.55	2	72.7666667	6.95277722	3	62.9	7.82285114	5	67.56	9.65352785	5
Cer d18:1/16:0	14498	104.6	2	11208.6333	2498.44069	3	11032.62	1589.02001	5	11074.58	2899.32353	5
Cer d18:1/18:0	1303.55	304.65	2	1092.43333	581.892946	3	1333.24	249.534247	5	1734.28	481.739203	5
Cer d18:1/20:0	552.9	160.4	2	399.966667	164.993458	3	691.26	76.5137935	5	773.24	270.078416	5
Cer d18:1/22:0	3220.65	188.65	2	2661.73333	884.146342	3	3397.44	301.261329	5	3318.06	750.118576	5

Cer d18:1/24:0	3793.4	573.8	2	3621.06667	1013.60006	3	5502.66	2039.51514	5	6274.94	2063.59935	5
Cer d18:1/24:1	19555.05	2328.65	2	19153.4333	4940.47842	3	17031.68	3293.38538	5	14291.3	2670.40225	5
HexCer d18:1/16:0	31169.3	1369.7	2	24764.9	7150.53702	3	**18072.36**	4144.8466	5	**14358.7**	3747.74596	4
HexCer d18:1/18:0	2779.9	320.6	2	1668.3	392.244171	3	1956.525	241.19419	4	1892.25	452.15855	4
HexCer d18:1/24:1	39567.8	2736.8	2	35489.9667	9702.39374	3	**29027.72**	3921.73571	5	**20640.18**	4714.63537	5
Hex2Cer d18:1/16:0	776.15	88.55	2	955.533333	94.7579783	3	891.275	134.471939	4	1186.2	190.323786	3
Hex2Cer d18:1/24:0	265	32.8	2	243.466667	72.3218808	3	417.64	141.256298	5	509.85	232.737243	4
Hex2Cer d18:1/24:1	1333.2	380.7	2	1787.36667	466.057651	3	2130.04	363.681909	5	1842	760.967499	4

Data to Supplement Figure S1B spleen

	WT Ctrl			LCK/Cre Ctrl			WT DSS			LCK/Cre DSS		
	Mean	SEM	N	Mean	SEM	N	Mean	SEM	N	Mean	SEM	N
SPH d18:1	1139.085	49.655	2	1410.38333	70.499558	3	1259.584	100.037302	5	1287.218	303.221966	5
SPH d18:0	300.33	25.02	2	352.446667	14.4759966	3	321.91	20.5306286	5	379.192	95.6068986	5
S1P d18:1	42.94	3.78	2	44.89	7.59399763	3	59.21	14.5288214	5	66.47	8.60881293	5
S1P d18:0	34.625	0.255	2	33.0833333	1.976439	3	39.18	5.31538522	5	33.608	3.67778656	5
Cer d18:0/16:0	383.1	0	1	315.033333	13.0966069	3	264.666667	6.8316258	3	374.4	51.5772721	4
Cer d18:0/18:0	124.1	0	1	107.9	6.58862656	3	79.2333333	9.6175765	3	89.76	10.5844981	5
Cer d18:0/24:0	303.1	47	2	347.4	33.1364351	3	398.82	51.2762655	5	540.4	53.2166985	5
Cer d18:0/24:1	868.1	0	1	779.833333	84.6559573	3	826.48	73.069586	5	**1349.06**	233.820304	5
Cer d18:1/14:0	26.4	10	2	17.6	2.25905585	3	16.1	0	1	16.975	0.6980628	4
Cer d18:1/16:0	7530.85	4577.55	2	5793.9	891.354236	3	**3287.82**	353.602249	5	4179.26	830.213086	5
Cer d18:1/18:0	1343.05	526.25	2	1062.2	82.6001816	3	656.08	108.864257	5	626.88	124.681095	5
Cer d18:1/20:0	1349.4	677.6	2	837	81.6413498	3	568.48	79.8061489	5	552.74	132.901485	5
Cer d18:1/22:0	3667.1	1149.5	2	3281.36667	264.81748	3	2661.88	233.604836	5	3377.76	584.578375	5
Cer d18:1/24:0	10353.6	6427.3	2	6622.16667	902.103457	3	**6228.34**	893.151077	5	7364.64	965.374381	5
Cer d18:1/24:1	16569.8	5717.4	2	15246.5667	556.662544	3	**11486.9**	1223.06298	5	11652.54	1849.19894	5
HexCer d18:1/16:0	11668.5	1200.1	2	9569.36667	2721.02621	3	9318.74	1074.42922	5	8252.1	1184.8775	5
HexCer d18:1/18:0	4102.7	558.3	2	2423.73333	471.90443	3	3219.3	297.384912	5	2817.56	532.753817	5
HexCer d18:1/24:1	35151.35	3593.15	2	**25388.3**	3219.48446	3	**28380.34**	4756.32482	5	**30924.98**	6036.29601	5
Hex2Cer d18:1/16:0	941.4	0	1	782.533333	69.1216882	3	889	110.473906	5	958.08	215.729744	5
Hex2Cer d18:1/24:0	1536.1	167.6	2	2086.03333	396.585452	3	2111.32	421.190134	5	2117.28	327.99845	5
Hex2Cer d18:1/24:1	3262.8	845.8	2	4832.9	742.071158	3	4063.12	1185.56351	5	5726.22	1176.70758	5

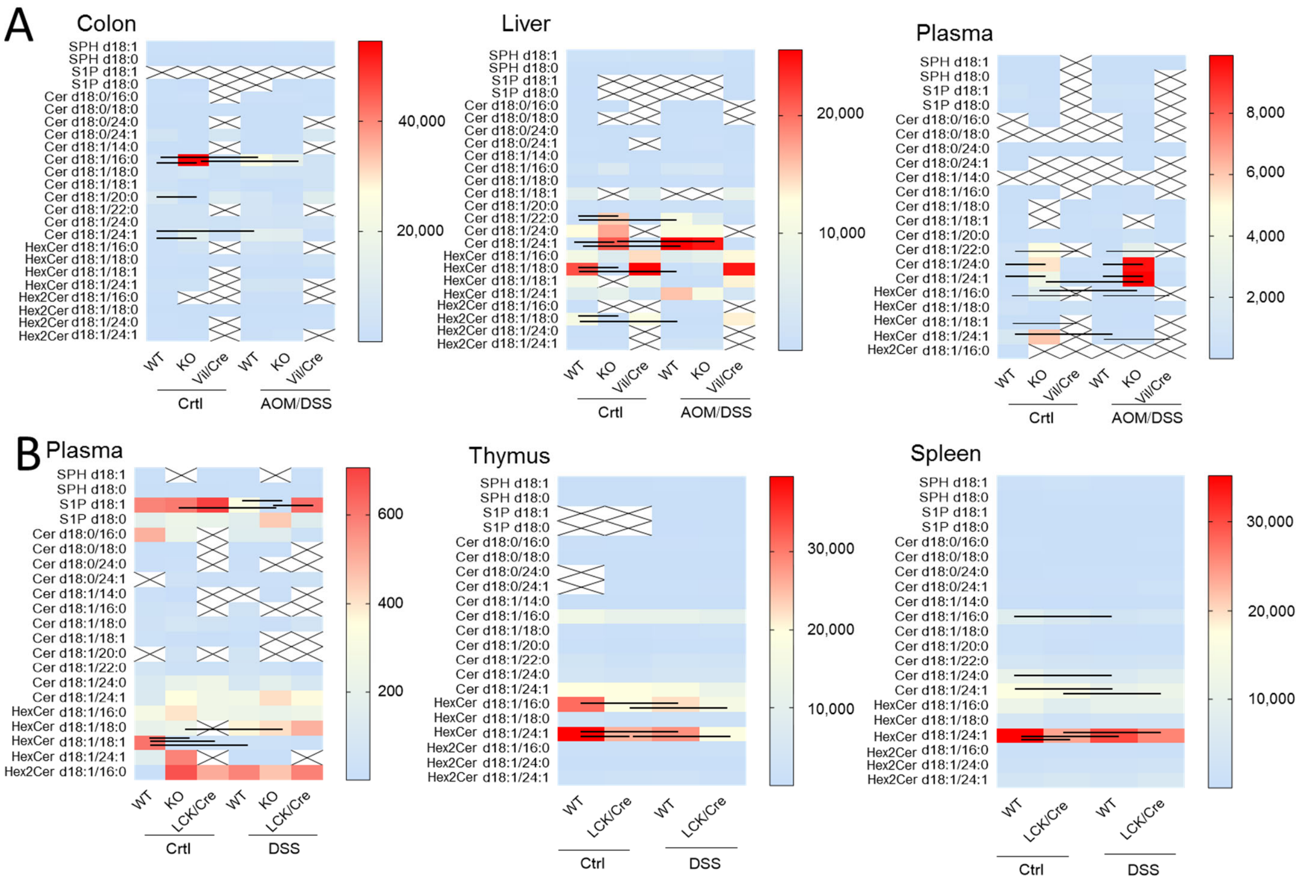

Figure S1. Sphingolipid status in different tissues after AOM/DSS and DSS treatment. In the heatmaps the median is presented and significant differences are indicated with lines between the groups. Cells with X indicates values which were under the detection limit of quantification. (**A**) CerS4 WT, KO, Vil/Cre of colon, liver and plasma. With the group sizes of WT Ctrl n=13, WT AOM/DSS *n* = 9–11, KO Ctr *n* = 3, KO DSS *n* = 3, Vil/Cre Ctrl *n* = 2, Vil/Cre DSS *n* = 4. (**B**) Sphingolipid measurements including CerS4 LCK/Cre tissue. Plasma, thymus and spleen measurements of DSS treated mice of CerS4 WT, KO and LCK/Cre mice WT Ctrl *n* = 6, WT DSS *n* = 11, KO Ctrl *n* = 7 KO DSS *n* = 6, LCK/Cre Ctrl *n* = 2, LCK/Cre DSS *n* = 5.

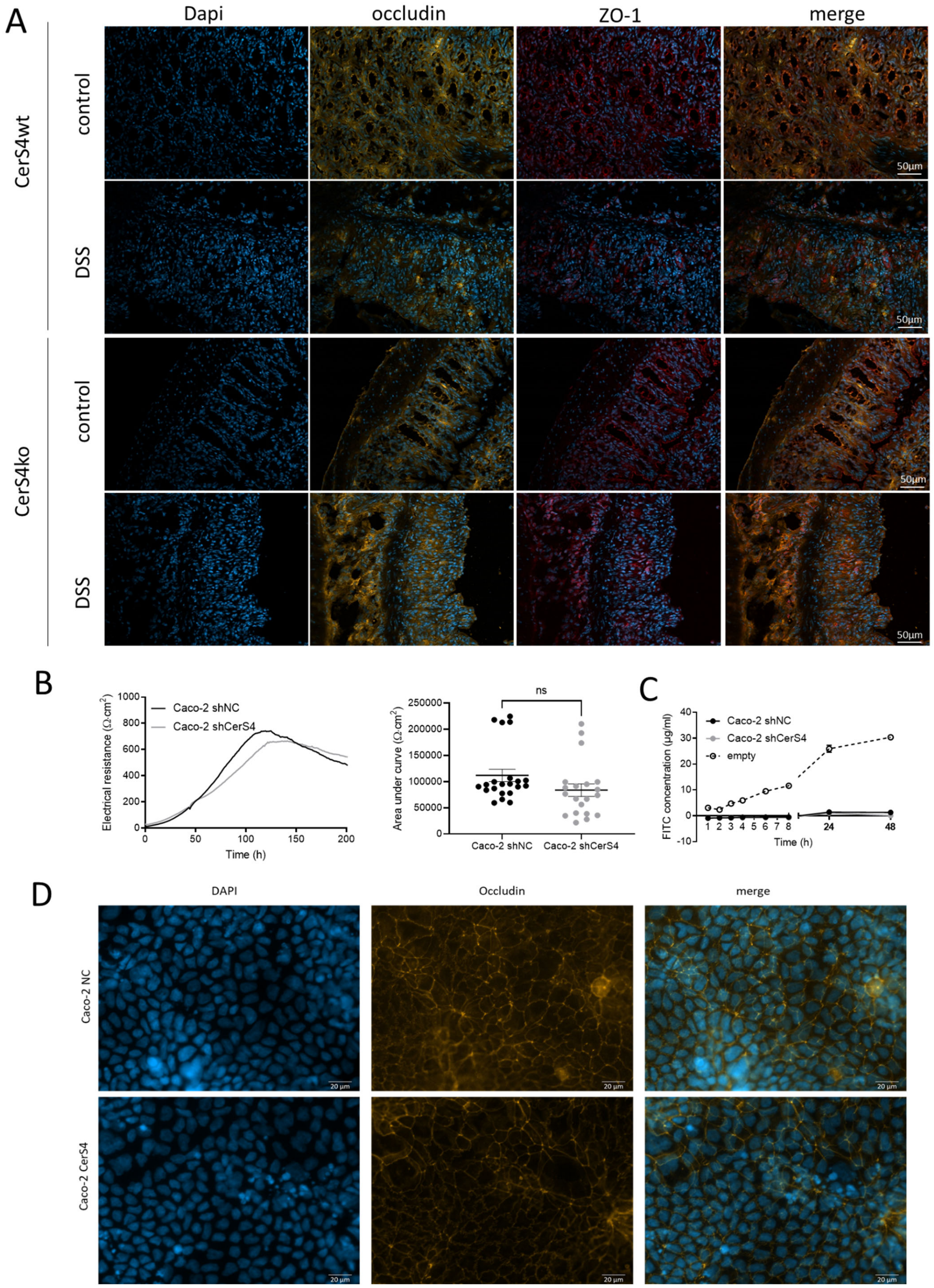
A
Dapi
occludin
ZO-1
merge
CerS4wt
control
DSS
CerS4ko
control
DSS
50µm
B
Electrical resistance (Ω·cm²)
Caco-2 shNC
Caco-2 shCerS4
Time (h)
Area under curve (Ω·cm²)
ns
Caco-2 shNC
Caco-2 shCerS4
C
FITC concentration (µg/ml)
Caco-2 shNC
Caco-2 shCerS4
empty
Time (h)
D
DAPI
Occludin
merge
Caco-2 NC
Caco-2 CerS4
20 µm

Figure S2. CerS4 deficiency does not influence tight junction protein expression in mice or barrier function in human Caco-2 cells. (**A**) Immunohistochemical staining of occludin (Cy3), ZO-1 (Alexa Fluor® 647) and Dapi in mouse tissue of CerS4 WT and CerS4 KO mice with and without DSS treatment.Picture were taken with a Zeiss Axio Imager Z1 microscppe with Apoptome unit. (**B**) Transepithelial electrical resistance (TEER) was measured in Caco-2 cells using a CellZscope2 instrument. Calculation of electrical resistance was performed by CellZscope software. Exemplary measurement of electrical resistance over time of Caco-2 control and CerS4 downregulated cells. Area under the curve of electrical resistance in mean ± SEM of $n = 3$ independent experiments. Statistical analysis was performed by Student's t-test. Permeability assay of fluorescein isothiocynate (FITC) dextran at 100 µg\ml (average size 40,000). Data are mean ± SEM of $n = 3$ independent measurements. Statistical analysis was performed by one-way ANOVA. (**C**) Immunocytochemical staining of occludin in Caco-2 cells after TEER measurement. (**D**) Cells were fixated with 4% PFA and stained with anti-occludin and secondary Cy3 anti-mouse antibody and 4′,6-diamidino-2′-phenylindoldihydrochloride (DAPI). 40x magnification taken with Axio Observer (Zeiss). Scale bar 20 µm.

Blood

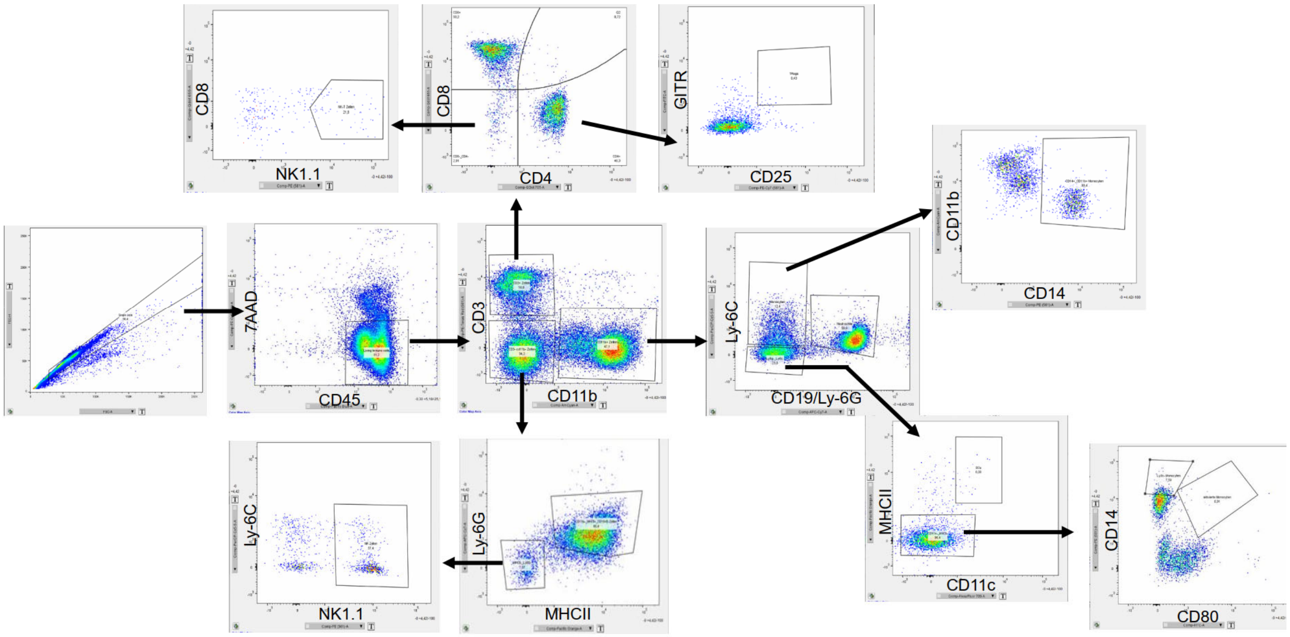

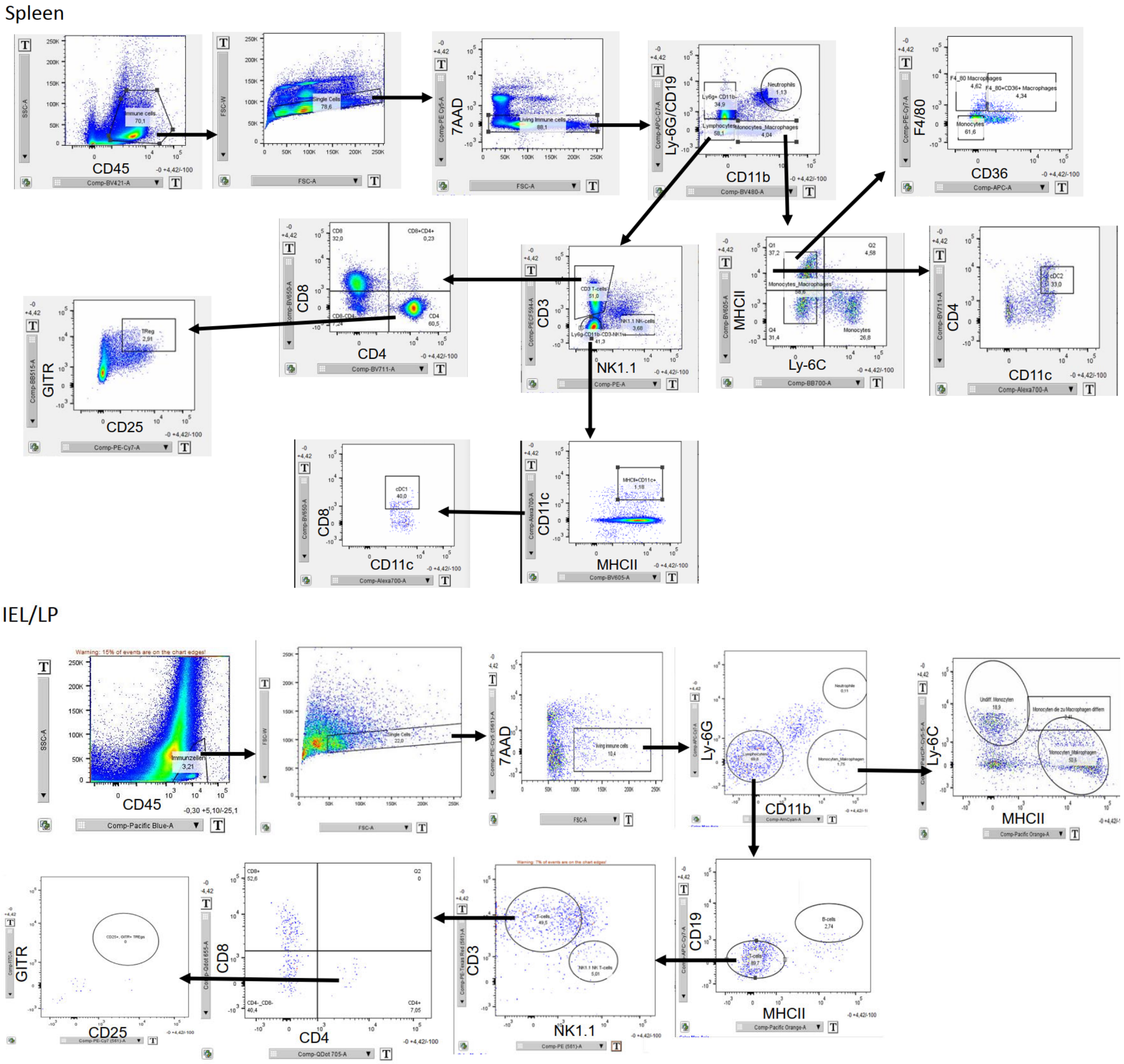

Figure S3. Gating strategy for FACS analysis. The gating strategies in blood, spleen and IEL/LP were quite comparable but with some changes. FACS measurement was performed with BD LSR-Fortessa™ Cell Analyzer (BD Bioscience, Heidelberg, Germany) and were analyzed using FlowJo software v10 (Treestar, Ashland,MA, USA).
